# Impact of therapeutic hypothermia on cardiogenic shock outcomes: a systematic review and meta-analysis

**DOI:** 10.1186/s13613-025-01541-0

**Published:** 2025-08-19

**Authors:** Miloud Cherbi, Bruno Levy, Paul Gautier, Nadia Aissaoui, Pierre-Grégoire Guinot, Hamid Merdji, Clément Delmas

**Affiliations:** 1 Cardiology Department, Toulouse University Hospital, Toulouse, France; 2 Réanimation Médicale Brabois, CHRU Nancy, Vandoeuvre-Les Nancy, France; 3 Service de Cardiologie, AP- HP, Hôpital Européen Georges Pompidou, Paris, France; 4 Department of Anesthesiology and Intensive Care, Dijon University Hospital, Dijon, France; 5 Medical Intensive Care Unit, CHU de Strasbourg, Strasbourg, France

**Keywords:** Hypothermia, Cardiogenic shock, Mortality, Meta-analysis, Randomized controlled trials, Pneumonia, Bleeding, Sepsis

## Abstract

**Background:**

Pre-clinical studies have suggested the benefits of therapeutic hypothermia in cardiogenic shock (CS). However, current evidence on its efficacy and safety in CS remains limited.

**Methods:**

We performed a systematic review and meta-analysis to assess efficacy/safety of hypothermia in CS. PUBMED/EMBASE/Cochrane/Scopus/Web of Science were searched from inception to December 31, 2024, for studies evaluating outcomes of hypothermia in CS. Efficacy outcome was all-cause mortality. Safety outcomes included pneumonia, sepsis, and bleeding.

**Results:**

Seven studies including 695 patients were analyzed. Acute myocardial infarction (AMI)-related CS was the primary etiology in 363 patients (52.2%). Hypothermia was not associated with a significant reduction in all-cause mortality at 30 days (OR 0.83 [0.54–1.26] or at the longest available follow-up (IRR 0.85 [0.72–1.01]). No significant differences were observed for pneumonia (OR 1.44 [0.42–4.87]), sepsis (OR 0.61 [0.01–46.80]), or bleeding (OR 1.36 [0.65–2.89]). Meta-regression suggested that hypothermia may be less beneficial and riskier in patients with AMI-CS, whereas greater benefit was observed in those with mechanical circulatory support. Trial sequential analysis indicated that the cumulative Z-curve for hypothermia did not cross the boundary for benefit, nor the futility boundary, suggesting that current evidence remains inconclusive and underpowered.

**Conclusion:**

In this meta-analysis, therapeutic hypothermia appeared safe but failed to show a significant reduction in all-cause mortality in patients with CS, albeit with very low certainty of evidence. Larger RCTs are warranted to clarify its clinical utility.

**Supplementary Information:**

The online version contains supplementary material available at 10.1186/s13613-025-01541-0.

## Introduction

Cardiogenic shock (CS) is a life-threatening hemodynamic condition due to primary cardiac dysfunction resulting in an inadequate cardiac output which can result in multi-organ failure (MOF) and death [1]. Despite advancements in pharmacologic and mechanical circulatory support (MCS), CS remains a major challenge for the medical community, with a poor prognosis and a one-month mortality rate of approximately 25–30% [2], due in part to its diverse etiologies and phenotypes requiring tailored treatment. As a result, the evidence supporting commonly used treatments for CS remains limited, with no clear consensus on best practices, except for early revascularization in acute myocardial infarction (AMI)-CS cases [1]. However, while growing evidence suggests that non-ischemic CS is more common in practice [2], it remains underrepresented in most randomized trials, which primarily focus on AMI-CS patients [3].

Besides, severe CS is associated with ischemia-reperfusion injury and a proinflammatory cytokine profile, potentially resulting in intense vasoplegia and microcirculatory dysfunction [4]. Consequently, therapeutic hypothermia has been proposed as an adjunct therapy in the management of CS, as it may help counteract these detrimental effects, improving cardiac contractility, and reducing metabolic rate [5]. While therapeutic hypothermia has been extensively studied and applied to patients after resuscitated cardiac arrest, its clinical efficacy in CS has yet to be validated. Despite promising preliminary results [6], recent studies failed to show clinical benefits [7–13], although most studies were underpowered, making it challenging to draw definitive conclusions about this intervention.

Therefore, we designed the present systematic review and meta-analysis aiming to assess the efficacy and safety of therapeutic hypothermia among patients with CS.

## Methods

### Protocol registration

The study was registered with PROSPERO, CRD42025631481. The meta-analysis was performed in line with recommendations from the Cochrane Collaboration and the Preferred Reporting Items for Systematic Reviews and Meta-Analyses (PRISMA) Statement (PRISMA checklist provided in Supplementary Table 1) [14, 15].

### Data sources & search strategy

Databases (PubMed, EMBASE, Web of Science, Scopus, and the Cochrane Central Register of Controlled Trials) were searched without language restrictions for both randomized clinical trials (RCTs) and non-randomized case-controlled studies assessing therapeutic hypothermia in settings of CS, using the search terms outlined in Supplementary Table 2, from inception to December 31, 2024. Searches of unpublished grey literature were performed manually on Google Scholar, OpenGrey, and pre-print servers (MedRxiv). ClinicalTrials.gov was also searched for ongoing studies on CS and hypothermia. The search was kept up to date by setting up automated alerts from PUBMED for new publications. Institutional review committee approval was not required given that this is a meta-analysis of published data.

### Eligibility criteria

We included studies comparing therapeutic hypothermia to standard care in CS patients, with no minimum sample size required. All therapeutic hypothermia protocols were included. Only articles published in peer-reviewed international journals were considered.

### Study selection

**Fig. 1:**
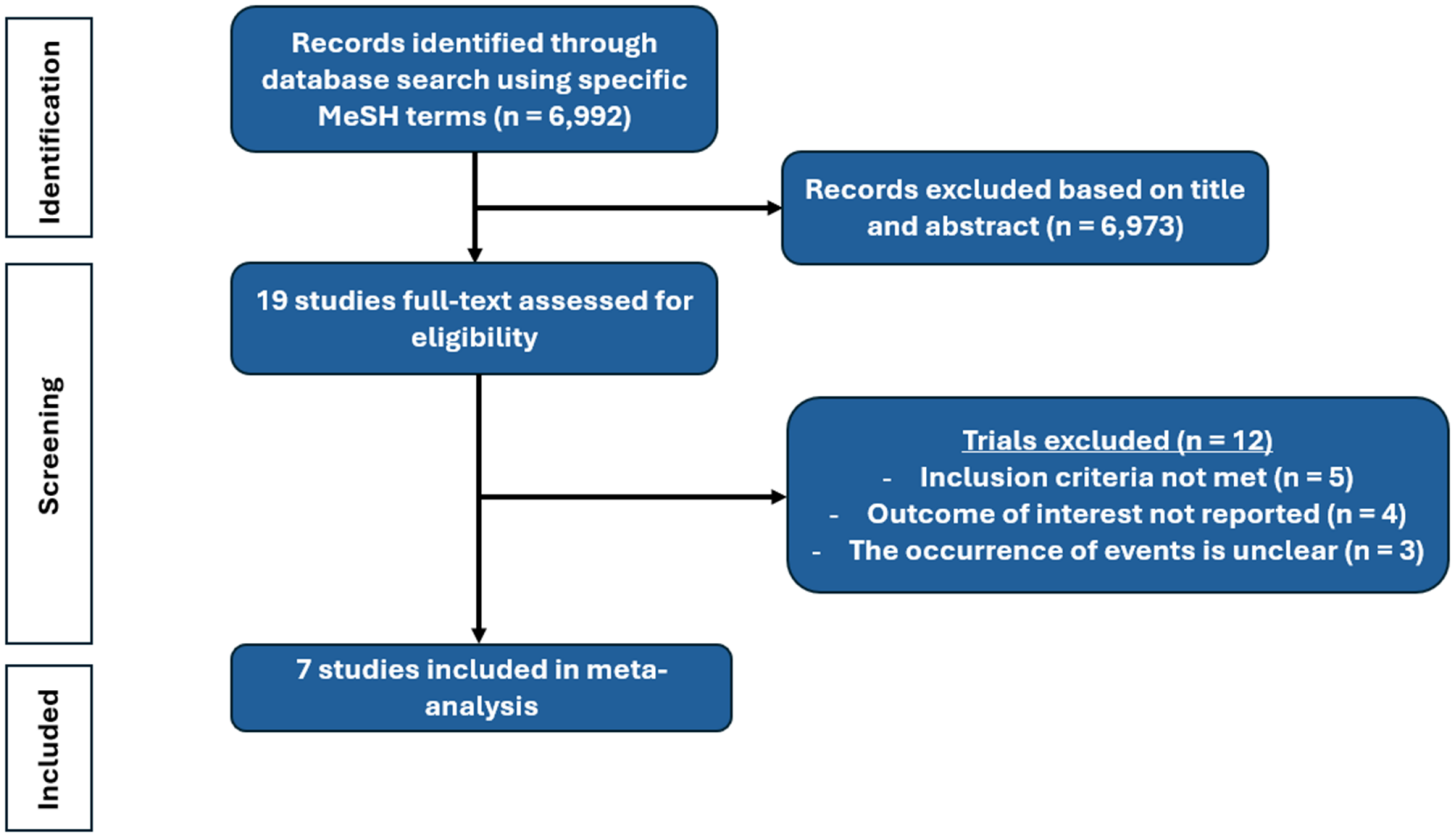
Study selection

Titles and abstracts of studies retrieved using the search strategy were independently screened by 2 investigators (C. D. and M. C.) to identify studies with an active arm involving therapeutic hypothermia in CS patients. The full texts of potentially eligible studies were retrieved and independently assessed for eligibility by the two investigators. Discrepancies between investigators were discussed collegially and the final decision was then made by consensus. The study selection process is illustrated in a PRISMA flow chart (Fig. 1).

### Outcomes

The primary outcome was 30-day all-cause mortality. Secondary outcomes included all-cause mortality at the longest reported follow-up, as well as the incidence of pneumonia, sepsis, bleeding, and supraventricular or ventricular arrhythmias. For each outcome, the definitions provided in the original studies were used in the analysis.

### Data extraction

Two authors (C.D. and M.C.) independently extracted data on study characteristics (including study ID, country, design, sponsor, inclusion/exclusion criteria, sample size, therapeutic hypothermia protocol, and follow-up period), baseline patient characteristics (such as sex, age, AMI-CS, post-cardiotomy CS, hypertension, ischemic cardiomyopathy, cardiac arrest, intubation, mechanical ventilation, and temporary MCS), and outcomes (all-cause and 30-day mortality, pneumonia, sepsis, bleeding, and arrhythmias).

### Risk of bias and certainty of evidence

For RCTs, the risk of bias was assessed using the Cochrane Risk of Bias 2 (RoB 2) tool [16], which evaluates five predefined domains: the randomization process, deviations from intended interventions, missing outcome data, measurement of the outcome, and selection of the reported results. For non-randomized observational studies, the risk of bias was assessed using the ROBINS-I tool [17], which considers seven domains: confounding factors, classification of interventions, selection of participants, deviations from intended interventions, missing data, measurement of outcomes, and selection of the reported results. Assessment for publication bias was limited due to the small number of RCTs included (< 10), especially for funnel plot and Egger’s test [18]. Reviewers (C.D and M. C.) used the GRADE (Grades of Recommendation, Assessment, Development and Evaluation) approach to assess the quality of evidence for each outcome [19]. GRADE appraises the confidence in estimates of effect by considering within-study risk of bias, directness of the evidence, heterogeneity of the data, precision of effect estimates, and risk of publication bias.

### Statistical analysis

A random-effects model was used to perform the meta-analysis since, based on preexisting clinical and pathophysiological considerations [7–13], we could not rule out the possibility that the observed estimates of hypothermia might vary across studies because of real differences in the treatment effect in each study as well as sampling variability. All the efficacy and safety outcomes studied were dichotomous. Hence, the number of events and total sample size in each group were extracted from each study for each outcome of interest. A pooled analysis was then conducted by aggregating the data to calculate the odds ratios (ORs) and corresponding 95% confidence intervals (CIs) for each dichotomous outcome. Between-study heterogeneity was estimated using the restricted maximum likelihood method. The Hartung-Knapp-Sidik-Jonkman correction was applied to derive more robust confidence intervals [20]. Statistical heterogeneity was assessed using multiple complementary measures. The I² statistic quantified the proportion of total variation attributable to between-study differences rather than chance, with I² ≥50% indicating substantial heterogeneity [14]. Since I² does not reflect the magnitude of effect size variation [21], we additionally reported: (1) between-study variance (τ²), reflecting variability in intervention effects across studies [14], and (2) 95% prediction intervals (PI) when ≥ 3 studies were available, representing the expected range of true effects in future similar studies and providing a comprehensive estimate of treatment effect variability [14, 22]. Eventually, in light of gaps in the evidence and previous studies [1, 7–13], a meta-regression analysis was conducted to assess the effect of AMI-CS, temporary MCS (Impella or intra-aortic balloon pump [IABP] or extracorporeal membrane oxygenation [ECMO]), and cardiac arrest on mortality.

The meta-analysis was performed using R software version 4.3.2 and the R packages *meta* and *metafor*.

### Trial sequential analysis

Given the limited number of included studies, we conducted a trial sequential analysis (TSA) to evaluate the reliability of our results, using a random-effects model for all-cause mortality, assuming a 5% significance level, 90% power, and a 10% mortality reduction as a clinically meaningful threshold [14]. The TSA was conducted for the primary outcome of 30-day all-cause mortality and for all-cause mortality at the longest reported follow-up to assess the robustness of our findings. A model variance-based heterogeneity correction was applied, and analyses were performed using TSA V.0.9.5.10 beta (Copenhagen Trial Unit, Rigshospitalet, Denmark). The TSA approach was employed to balance type I and type II errors, providing an estimate of the required information size indicating the effect size would be substantial enough to withstand the impact of additional studies [23, 24]. Trial sequential monitoring boundaries were drawn, similar to interim analysis of randomized trials, and provide information as to whether to continue evaluating for evidence when the boundary is not crossed or whether sufficient evidence is reached for an anticipated effect or for futility when the boundary is crossed. The interpretation is similar to that of DeMets stopping boundaries for clinical trials. Crossing the TSA boundary indicates sufficient evidence for reliable conclusions, while failure to do so suggests the need for further research.

### Sensitivity analysis

To assess the robustness of the main analysis and account for different follow-up durations between studies, a first sensitivity analysis was performed using the incident rate of outcomes per person-years to obtain the incidence rate ratios (IRRs) of therapeutic hypothermia relative to control using a random-effects model with restricted maximum likelihood method estimation of between-study heterogeneity. Person-years of follow-up were calculated by multiplying the sample size with the average (mean/median) follow-up duration. Additionally, three other sensitivity analyses were performed: [1] excluding the HYPO-ECMO trial, which included the majority of patients, to assess the robustness of the results; [2] restricting the analysis to studies with a low risk of bias; [3] restricting the analysis to studies applying a therapeutic hypothermia protocol with a target temperature of 32–34 °C maintained for 24 h.

## Results

### Baseline characteristics

From 6,992 references retrieved by searching relevant online databases, 19 studies were selected for full-text assessment of inclusion criteria based on title and abstract screening. Flow chart of literature retrieval and reasons for article exclusion are shown in Fig. 1. Finally, 7 studies could be included in our study [7–13], that included a total of 695 patients. Among them, 4 (SHOCK-COOL [10], Fang et al. [11], HYPO-ECMO [12], CHILL-SHOCK [13]) were RCTs while the remaining three (Zobel et al. [7], Orban et al. [8], Blatt et al. [9]) were case-controlled studies. All interventional groups targeted a temperature range of 32 to 34 °C, except for the study by Fang et al. [11], which aimed for a target of 34 to 35 °C. In six out of seven studies, the targeted temperature was maintained for 24 h, except for the study by Blatt et al. [9], where the duration was 12 h. Nearly all patients in the therapeutic hypothermia groups achieved the targeted temperature (571 out of 580 patients; data not reported for the remaining 115). All control arms aimed to achieve normothermia. The vast majority of included patients (*N* = 500/510; 98.0%, with two studies missing data) were intubated, sedated, and receiving invasive mechanical ventilation. The main elements of the protocol and the characteristics of the studied populations are presented in Table 1 and Supplementary Table 3.

**Table 1:** Baseline characteristics of the patients included in the meta-analysis

	Zobel et al.	Orban et al.	Blatt et al.	SHOCK-COOL	Fang et al.	HYPO-ECMO	CHILL-SHOCK
N^o^ patients enrolled	40 patients	145 patients included between January 2009 and May 2012	21 patients included in 2014	40 patients included between July 2012 - March 2015	95 patients included between January 2011 - December 2014	334 patients included between October 2016 - July 2019	20 patients included between November 2017 - March 2021
Age, years*	Hypothermia: 59.0 Control: 61.0	Hypothermia: 69.1 (13.0) Control: 70.4 (12.1)	Hypothermia: 69.9 (7.0) Control: 65.1 (10.0)	Hypothermia: 77.0 (8.0) Control: 76.0 (9.0)	Hypothermia: 44.5 (9.6) Control: 46.6 (7.2)	Hypothermia: 57 (12.0) Control: 59 (12.0)	Hypothermia: 62.2 (10.8) Control: 63.6 (10.3)
Male sex, n (%)	Hypothermia: 16 (80.0) Control: 17 (85.0)	Hypothermia: 55 (85.9) Control: 53 (65.4)	15 (71.4) Hypothermia: 6 (75.0) Control: 9 (69.2)	26 (65.0) Hypothermia: 12 (60.0) Control: 14 (70.0)	55 (57.9) Hypothermia: 29 (60.4) Control: 26 (55.3)	253 (75.7) Hypothermia: 128 (76.0) Control: 125 (75.0)	14 (70.0) Hypothermia: 7 (70.0) Control: 7 (70.0)
AMI-CS, %	31 (77.5) Hypothermia: 16 (80.0) Control: 15 (75.0)	145 (100.0)	21 (100.0)	40 (100.0)	0 (0.0)	121 (36.2) Hypothermia: 81 (48.0) Control: 78 (47.0)	5 (25.0) Hypothermia: 1 (10.0) 4 (40.0)
Post-cardiotomy CS, n (%)	NA	0 (0.0)	0 (0.0)	0 (0.0)	95 (100.0)	50 (15.0) Hypothermia: 23 (14.0) Control: 27 (16.0)	NA
Hypertension, n (%)	21 (52.5) Hypothermia: 12 (60.0) Control: 9 (45.0)	119 (82.1) Hypothermia: 53 (82.8) Control: 66 (81.5)	16 (72.6) Hypothermia: 5 (62.5) Control: 11 (84.6)	31 (77.5) Hypothermia 13 (65.0) Control: 18 (90.0)	NA	121 (37.1) Hypothermia: 61 (37.0) Control: 60 (37.0)	20 (100.0)
Ischemic cardiomyopathy, n (%)	31 Hypothermia: 15 (75.0) Control: 16 (80.0)	30 (20.7) Hypothermia: 17 (26.6) Control: 13 (16.0)	9 (42.9) Hypothermia: 3 (37.5) Control: 6 (46.2)	10 (25.0) Hypothermia: 7 (35.0) Control: 3 (15.0)	NA	72 (22.4) Hypothermia: 31 (19.0) Control: 41 (26.0)	9 (45.0) 4 (40.0) 5 (50.0)
Cardiac arrest, n (%)	40 (100.0)	Hypothermia: 61 (95.3) Control: NA	0 (exclusion criteria)	0 (exclusion criteria)	0 (0.0)	159 (47.6%) Hypothermia: 81 (48.0) Control: 78 (47.0)	NA
Intubation and mechanical ventilation, n (%)	NA	NA	21 (100.0)	40 (100.0)	95 (100.0)	334 (100.0)	10 (50.0) Hypothermia: 3 (30.0) Control: 7 (70.0)
Temporary MCS, n (%) ECMO IABP Impella	NA	NA	0 (0.0) 0 (0.0) 0 (0.0) 0 (0.0)	3 (7.5) 3 (7.5) 0 (0.0) 0 (0.0)	0 (0.0) 0 (0.0) 0 (0.0) 0 (0.0)	334 (100.0) 334 (100.0) NA NA	9 (45.0) 0 (0.0) 8 (40.0) 1 (5.0)

*Age is presented as mean (SD; Orban et al., HYPO-ECMO and CHILL-SHOCK) or median (IQR; Zobel et al., Blatt et al., SHOCK-COOL, Fang et al.)

AMI-CS, acute myocardial infarction related cardiogenic shock; CS, cardiogenic shock; ECMO, extracorporeal membrane oxygenation; IABP, intraaortic balloon pump; MCS, mechanical circulatory support; NA, not available

### Risk of bias

**Fig. 2:**
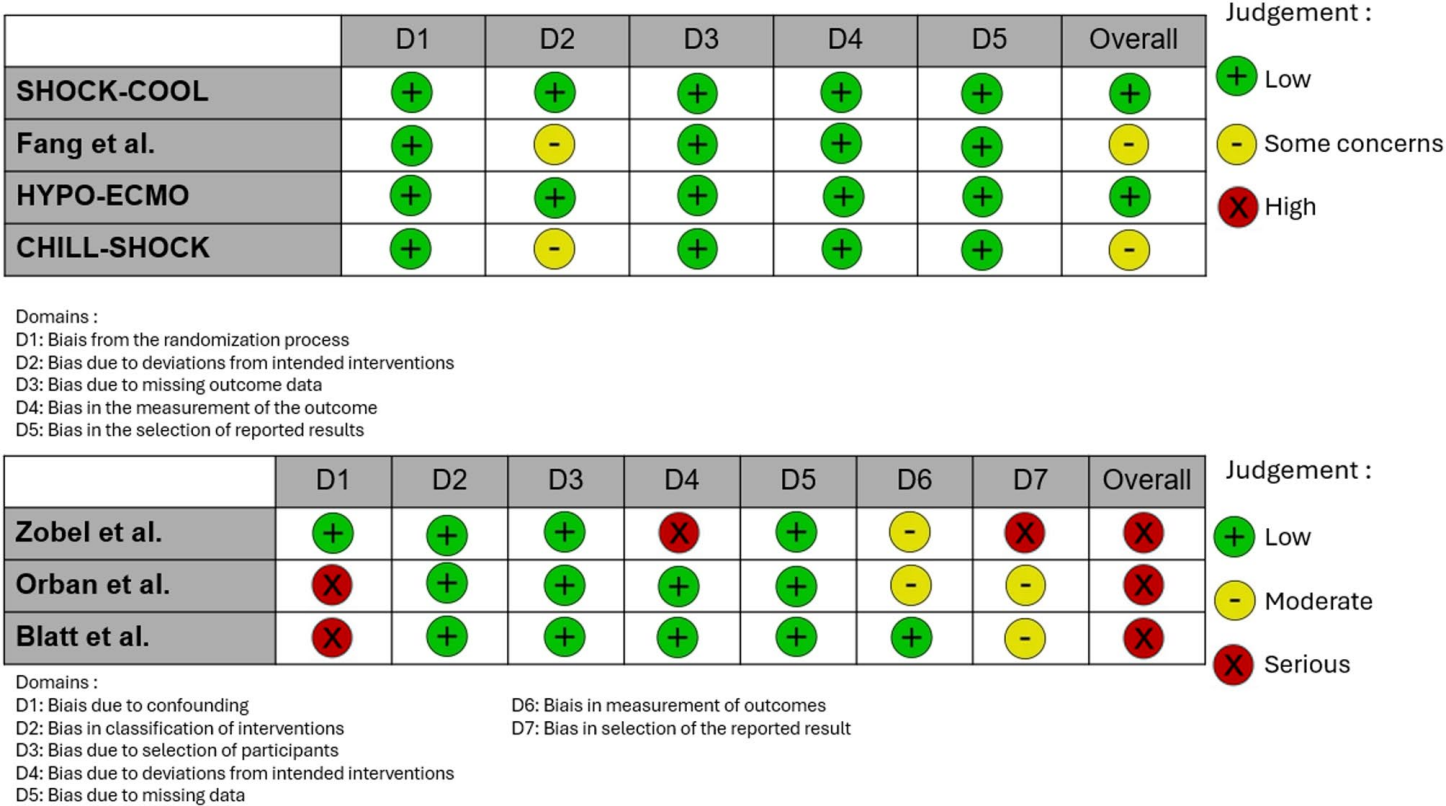
Risk of bias assessment using the RoB2 and ROBINS-I tools Upper panel shows risk of bias assessment for randomized controlled trials using the Cochrane Risk of Bias 2 (RoB 2) tool across five domains. Lower panel shows risk of bias assessment for observational studies using the ROBINS-I tool across seven domains

According to RoB2, the risk of bias analysis classified two included RCTs (SHOCK-COOL [10], HYPO-ECMO [12]) as having a “low” risk of bias across the five evaluated domains, whereas the study by Fang et al. [11] and CHILL-SHOCK [13] were classified as having “some concerns” due to insufficient detail regarding the hypothermia and rewarming protocol (Fig. 2, Supplementary Table 3). In contrast, the non-randomized studies (Zobel et al. [7], Orban et al. [8], and Blatt et al. [9]) were assessed as having a high risk of bias according to the ROBINS-I tool.

### Meta-analysis results

**Fig. 3:**
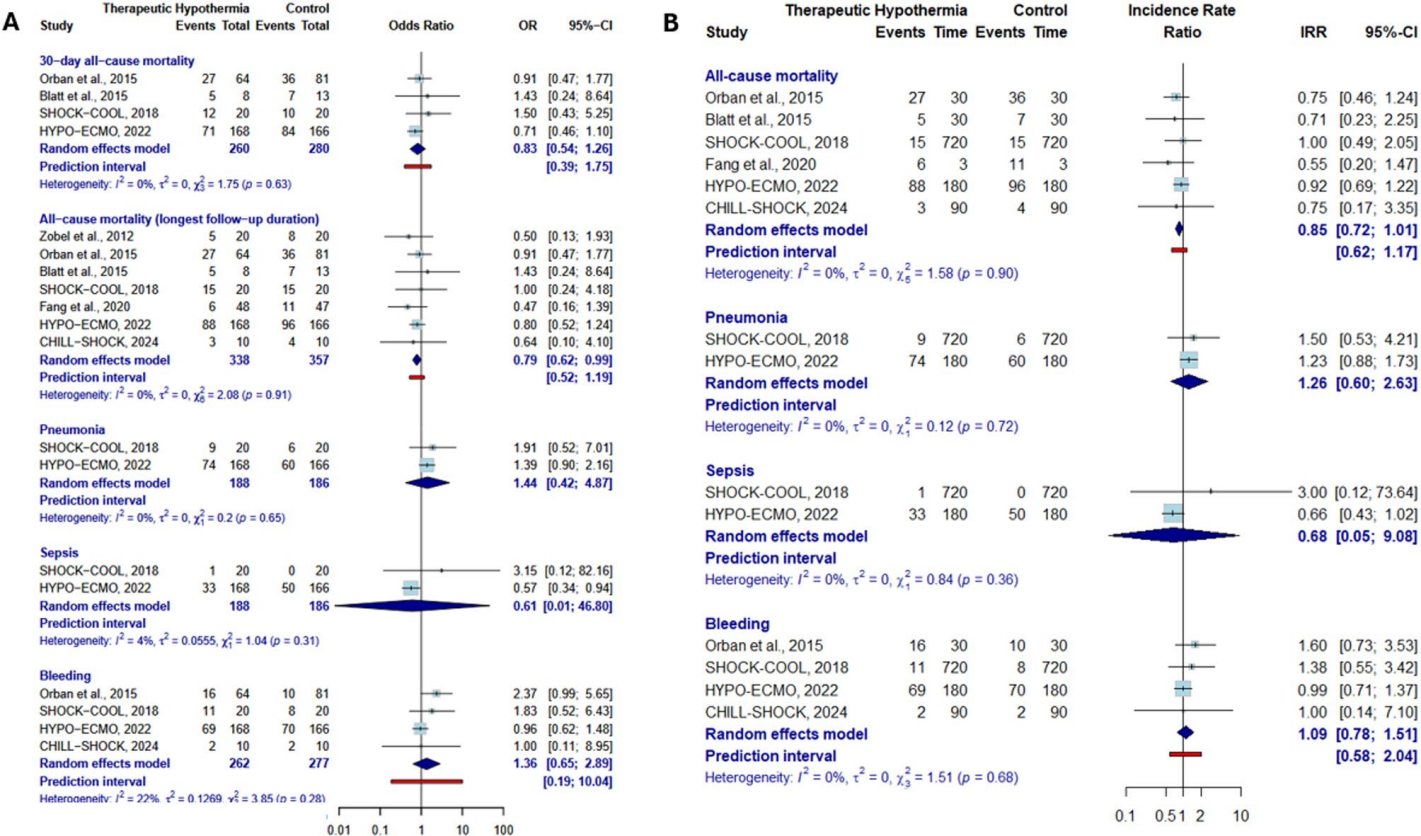
Meta-analysis of efficacy and safety endpoints for hypothermia in cardiogenic shock patients. A represented the main analysis. B represents the sensitivity analysis conducted using the incidence rate of outcomes per person-years to account for varying follow-up durations across the trials CI, confidence interval; IRR, incident rate ratio; OR, odds ratio

Overall, 338 patients received treatment with therapeutic hypothermia, while 357 were in the control group receiving standard care alone. Initial cardiac arrest was reported in 162 patients (49.4%) in the hypothermia group, with one study lacking this specific data [13]. AMI-CS was the primary etiology for CS onset in 363 patients (52.2%). Figure 3A shows that therapeutic hypothermia was not associated with a significant reduction in all-cause mortality at 30 days (OR 0.83 [0.54–1.26], I² = 0%, τ² = 0, PI 0.39–1.75). Considering the longest reported follow-up duration, therapeutic hypothermia was associated with a modest reduction in all-cause mortality (OR 0.79 [0.62–0.99]), with low heterogeneity (I² = 0%, τ² = 0), although the PI included both potential benefit and neutrality (PI 0.52–1.19). Conversely, sensitivity analysis adjusted for follow-up duration revealed the absence of a statistically significant reduction (IRR 0.85 [0.72–1.01]) (Fig. 3B). Furthermore, no difference was observed in the rates of pneumonia (OR 1.44 [0.42–4.87]), sepsis (OR 0.61 [0.01–46.80]), or bleeding (OR 1.36 [0.65–2.89]), as confirmed by the corresponding IRRs. The rates of arrhythmia, bradyarrhythmia, and ventricular arrhythmia described in each study were similar between the groups, but the definitions were not sufficiently consistent to allow for a pooled meta-analysis.

**Fig. 4:**
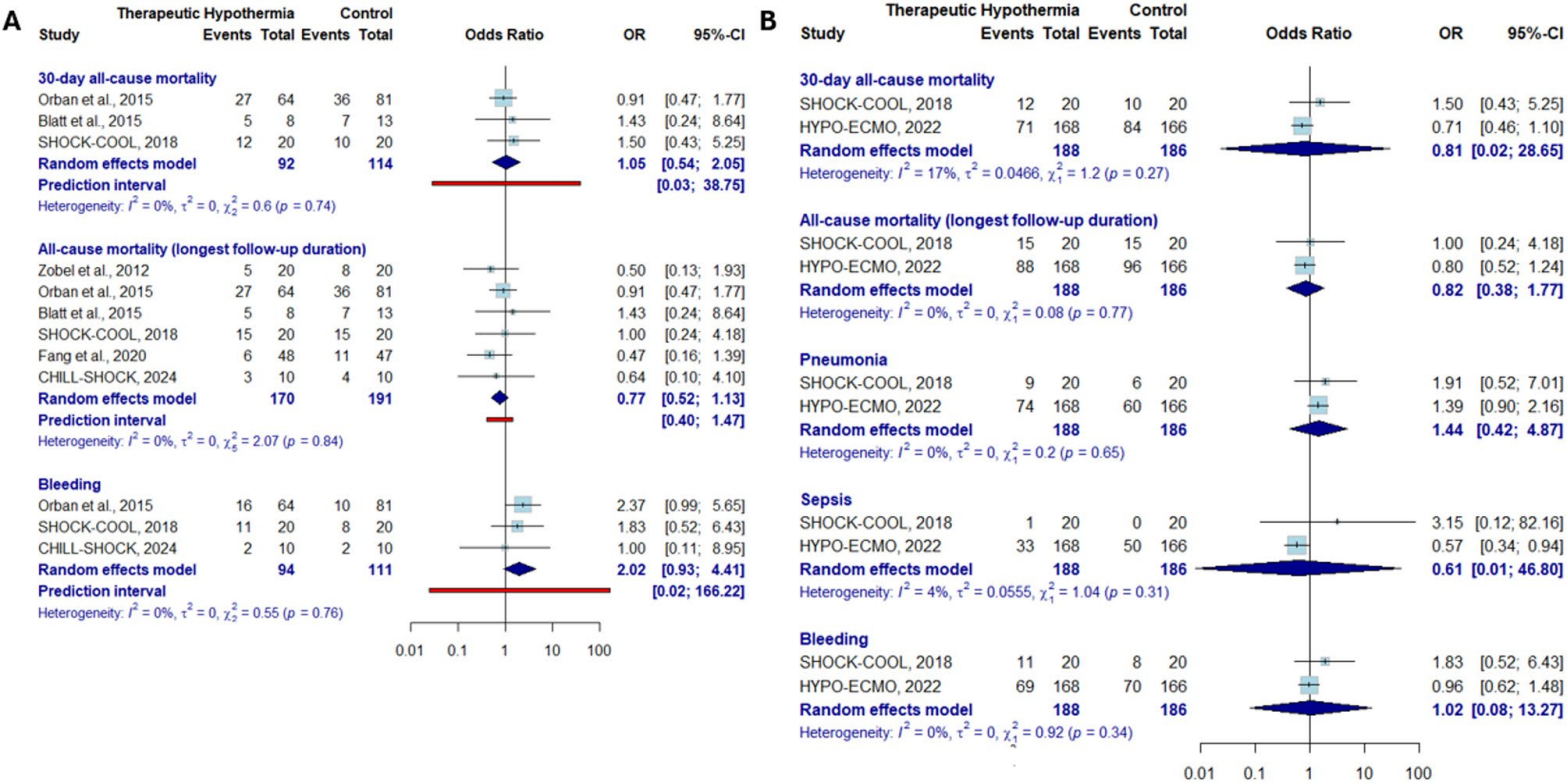
Sensitivity analysis of efficacy and safety endpoints for hypothermia in cardiogenic shock patients.

The first sensitivity analysis, which excluded the HYPO-ECMO trial—the largest contributor to the patient population—showed no significant effect on all-cause mortality (OR 0.77 [0.52–1.13]) (Fig. 4A). In the second sensitivity analysis, restricted to trials with a low risk of bias, no difference was observed for any of the efficacy or safety outcomes (Fig. 4B). Finally, the sensitivity analysis restricted to studies applying hypothermia between 32 and 34 °C for 24 h also yielded results consistent with the main analysis (Supplementary Fig. 1).

Meta-regression analyses revealed differential effects of patient characteristics on therapeutic hypothermia efficacy. Higher rates of AMI-CS were associated with reduced hypothermia effectiveness, with significant increases in 30-day mortality risk (OR 1.83 [1.01–3.35], *p* = 0.05) and bleeding complications (OR 3.57 [2.55-5.00], *p* < 0.01) (Supplementary Table 4). Conversely, increased temporary MCS utilization was associated with enhanced hypothermia benefits, showing improved 30-day survival (OR 0.48 [0.44–0.51], *p* < 0.01) and reduced bleeding risk (OR 0.54 [0.37–0.77], *p* < 0.01). A similar trend was observed in the ECMO-only subgroup, although the reduction in bleeding risk did not reach statistical significance (*p* = 0.09). Lastly, the proportion of cardiac arrest patients did not significantly impact all-cause mortality, whether at 30 days or at the longest available follow-up.

### TSA findings

**Fig. 5:**
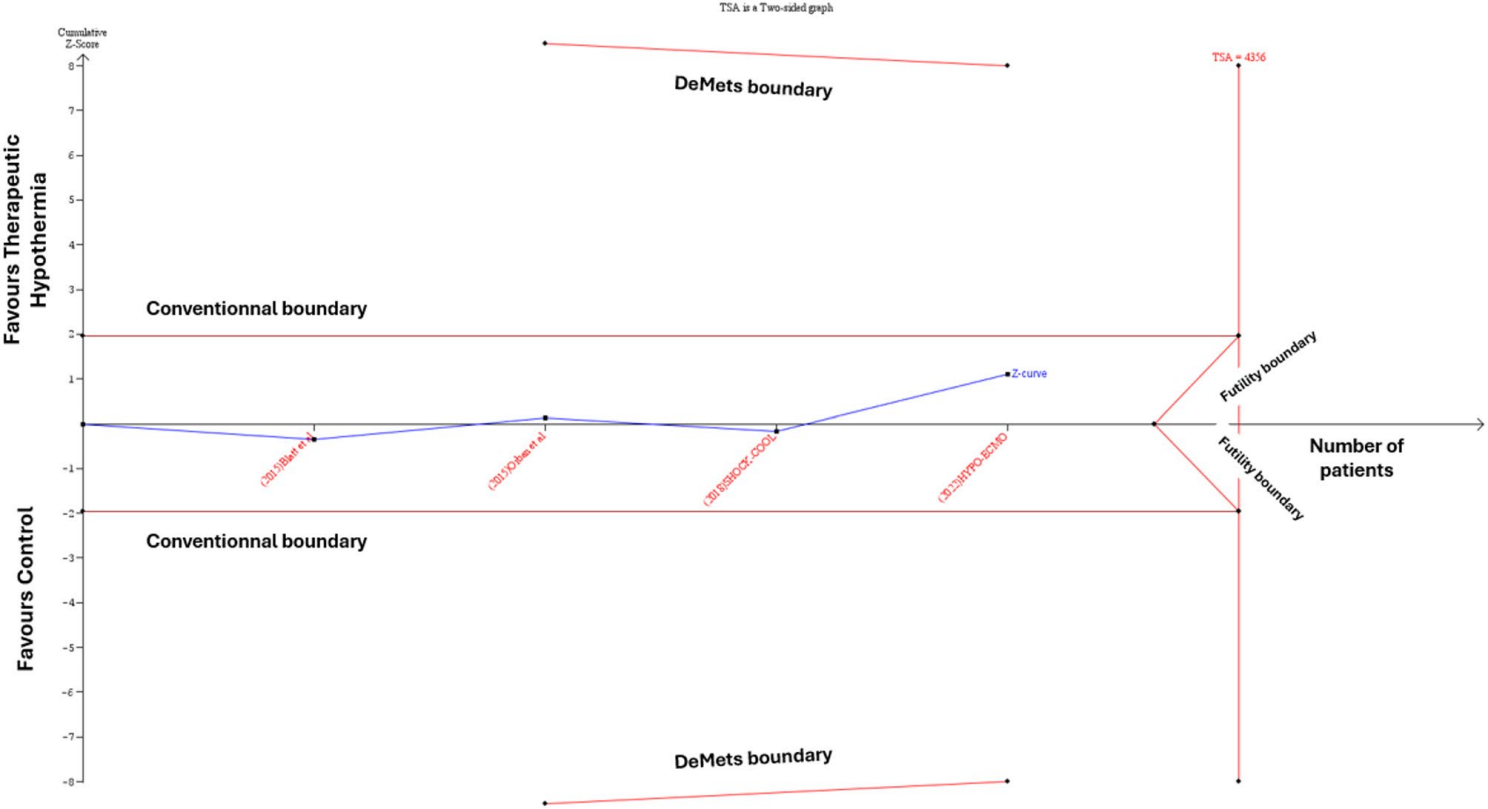
Trial sequential analysis of 30-day all-cause mortality outcome. The blue Z-curve represents the cumulative evidence from included studies plotted against the cumulative sample size. The horizontal red lines represent conventional boundaries for statistical significance (α = 0.05), while the slanted red lines show the trial sequential monitoring boundaries (DeMets boundaries) and futility boundaries. The required information size was calculated as 4,356 patients assuming a 10% relative risk reduction, 5% type I error, and 90% power

TSA for the primary outcome of 30-day all-cause mortality showed no statistically significant difference between therapeutic hypothermia and control groups, as the Z-curve did not cross the conventional boundary (Fig. 5). The Z-curve also did not cross the futility boundary or reach the required information size (*n* = 4356), indicating that the current evidence is underpowered to detect or exclude a clinically relevant difference. Similarly, TSA for all-cause mortality at the longest follow-up confirmed the absence of a statistically significant benefit, with the Z-curve crossing neither the conventional nor futility boundaries and not reaching the required information size (Supplementary Fig. 2). Both analyses indicate that current evidence is insufficient to demonstrate a benefit of therapeutic hypothermia in CS patients, and substantially more data would be needed to draw definitive conclusions.

### GRADE framework results

The GRADE system classified the evidence for all-cause mortality (both longest duration follow-up and 30-day) and bleeding as very low quality, while the evidence for other outcomes (pneumonia, sepsis) was classified as low quality. Further details and explanations are provided in Supplementary Table 5.

## Discussion

We present here the largest meta-analysis assessing the potentials benefits of therapeutic hypothermia in CS patients. Our main findings indicate that: (1) Based on currently available data, therapeutic hypothermia is not associated with a statistically significant reduction in all-cause mortality in CS, although the certainty of evidence is very low; (2) safety appears acceptable, as none of the adverse events—including pneumonia, sepsis, or bleeding—occurred more frequently with hypothermia; (3) the effect of hypothermia appears heterogeneous across different etiologies of CS, with a lower beneficial effect and a higher risk of bleeding observed in AMI-CS; and (4) despite a strong physiological rationale and encouraging preclinical data, prospective clinical studies remain scarce, and further research is still warranted to clarify the potential role of hypothermia in this setting.

Despite advances in pharmacological treatments and enhanced MCS, the mortality rate in CS remains unacceptably high, with no substantial improvement in recent years [25]. Several factors may explain this ongoing issue, including the variety of underlying etiologies and phenotypes leading to CS [1, 2], each requiring a tailored approach, as well as the low level of evidence supporting treatments that are nonetheless used daily. Moreover, there are still many unknowns regarding the complex pathophysiological mechanisms involved in the genesis of CS [26].

In this context, much hope has been placed in the use of therapeutic hypothermia, based on several potential benefits demonstrated in pre-clinical data. When applied to isolated myofibrils and cross-circulated hearts, mild hypothermia has been shown to improve myocardial contractility [27]. In vivo, hypothermia has been found to enhance stroke volume and cardiac output, likely mediated by an increased sensitivity of myofilaments to existing Ca2+, without a corresponding rise in myocardial oxygen consumption [28]. Finally, in dog- and porcine-based models of CS due to ischemia, therapeutic hypothermia has demonstrated improvements in hemodynamic and metabolic parameters, as well as reductions in mortality [29]. Despite these promising properties, the indications for therapeutic hypothermia have gradually declined over the past decade, particularly in the context of cardiac arrest, where it has been extensively studied, culminating in the results of the TTM2 trial [30], which found no difference between hypothermia (33 °C) and normothermia (< 37.8 °C). Similarly to cardiac arrest, one could hypothesize that, beyond achieving hypothermia, it might be the prevention of hyperthermia—which could exacerbate metabolic demand—rather than the attainment of strict hypothermia, that could also represent the primary therapeutic target in CS. Yet, in a post-hoc analysis of the HYPO-ECMO trial—the largest trial to date assessing therapeutic hypothermia in CS—focusing on ECMO-supported CS patients with frequent cardiac arrest prior to ECMO [31], hypothermia showed potential for reducing mortality without an increased risk of severe bleeding or infection. Besides, hypothermia appears to be relatively safe in our study, without an increase in bleeding, pneumonia, or sepsis, albeit with a small number of patients included. While data regarding hypothermia’s safety in CS patients are scarce, similar studies conducted in the context of cardiac arrest also indicate that it is not associated with an increased rate of adverse events [32]. Furthermore, the potential benefits of therapeutic hypothermia may be mitigated by adverse effects associated with invasive mechanical ventilation and sedation, as therapeutic hypothermia is poorly tolerated in conscious patients due to the risk of significant discomfort and shivering, which exacerbate VO_2_ and MVO_2_. Notably, 98% of the patients included in our meta-analysis were sedated, intubated, and mechanically ventilated. However, these results should be interpreted with caution in the context of CS, not only due to the small number of studies and patients included, but also because of the poor performance of RCTs in detecting adverse events, as they are generally not specifically designed for this purpose [33].

In our main analysis, therapeutic hypothermia failed to show a significant reduction in 30-day all-cause mortality. Similarly, no improvement in overall survival was observed when considering the longest follow-up duration in sensitivity analyses or after adjustment using incidence rate ratios. Interestingly, meta-regression analysis also suggested that the effect of hypothermia may vary depending on the CS profile, with a weaker survival benefit and higher risk of bleeding in AMI-related CS, and conversely, a greater survival benefit and lower bleeding risk in CS managed with temporary MCS. Of course, all these findings must be interpreted cautiously, given the limited number of studies and patients included. Nevertheless, they offer valuable insights that warrant further investigation in future studies. Indeed, CS is now recognized as a multifaceted condition with a broad range of etiologies [1, 2], each potentially driven by distinct and time-dependent inflammatory mechanisms. Thus, evaluating the potential benefit of therapeutic hypothermia in a more individualized manner could be valuable. Indeed, while CS secondary to AMI may benefit from early revascularization [1], other causes such as acute-on-chronic heart failure are not amenable to such a rapid and specific intervention, suggesting that the optimal duration of therapeutic hypothermia may need to be tailored to the underlying cause of CS. Furthermore, growing evidence suggests that inflammation may contribute to the maintenance and exacerbation of CS [34]. Given that hypothermia has demonstrated significant anti-inflammatory properties [35], the inflammatory CS phenotype -including cases related to autoimmune disease or myocarditis- could be a subgroup of interest for future evaluation of hypothermia’s potential benefit. Similarly, the same applies to mixed cardiogenic/septic shock, in which primary cardiac dysfunction is exacerbated by sepsis-induced and inflammation-mediated microcirculatory dysfunction, which could theoretically be amenable to treatment with hypothermia [36]. Conversely, several concerns have emerged regarding specific cases, such as congestive heart failure [37] or post-off-pump coronary artery bypass graft surgery [38], where hypothermia could have a detrimental effect. Our meta-analysis deliberately included all CS etiologies regardless of the underlying cause. However, given the highly heterogeneous nature of CS—both in terms of etiology and phenotype—future studies could focus on the potential benefits of hypothermia applied in specific subpopulations that may derive the greatest advantage, and further research is still needed to determine the optimal hypothermia protocol in CS, in which the current level of evidence remains very low.

Of course, the lack of observed survival benefit of hypothermia in CS patients may also result from insufficient statistical power, due to the limited number of studies conducted and the small sample sizes within each. In this regard, our trial sequential analysis—where the Z-curve crossed neither the conventional nor the futility boundaries, nor did it reach the required information size—suggests that further studies are still warranted to clarify the potential benefits of therapeutic hypothermia. Moreover, a Bayesian reanalysis of the HYPO-ECMO trial [10] revealed that, across a range of prior assumptions regarding the probability of benefit, the posterior probability of any mortality reduction (OR < 1) with moderate hypothermia ranged from 85 to 100%. Consequently, the authors concluded that the initial trial likely lacked sufficient statistical power to detect a moderate treatment effect that could still be clinically relevant.

Overall, our study illustrates that, although hypothermia has shown encouraging results in preclinical CS studies, there is currently insufficient data to support its effectiveness in clinical practice. However, at this stage of research, it is still too early to formally conclude its inefficacy, given the limited number of studies conducted, as highlighted by the results of the trial sequential analysis. Hence, further large-scale randomized clinical trials are still warranted to draw definitive conclusions, possibly considering the numerous etiologies and phenotypes of CS, for which the expected benefit of hypothermia may vary.

### Limitations and future directions

There was some heterogeneity in the application of therapeutic hypothermia across studies, including differences in the timing of initiation, cooling methods (external surface devices, intravascular catheters, ECMO circuit adjustment, or cold fluid infusion), and duration. While most studies used controlled systems with continuous core temperature monitoring and feedback-regulated devices, the methods varied across trials. Hypothermia was maintained for 24 h in six studies, while one study (Blatt et al.) applied it for only 12 h. Only one study (Fang et al.) targeted a temperature range outside of 32–34 °C, aiming instead for 34–35 °C. Data on the exact time to reach the target temperature and on rewarming protocols were inconsistently reported, which limits the ability to fully assess the impact of cooling and rewarming rates on outcomes. Nevertheless, results from the sensitivity analysis restricted to studies applying a 24-hour hypothermia protocol with a 32–34 °C target were consistent with the main analysis. Moreover, preclinical studies [27, 28] suggest that although hypothermia induces significant physiological changes compared to normothermia, hemodynamic differences between various hypothermic targets are not consistently observed. Eventually, there were significant differences in the usage rates of temporary MCS, ranging from 0 to 100% across the studies. Since available data on this topic is particularly scarce, we cannot exclude the possibility that the overall effect may have been influenced by potential confounding factors related to the specifics of each temporary MCS device, although these devices were evenly distributed across each arm. None of the included studies reported detailed information on withdrawal of life-sustaining therapy protocols, which limits the ability to account for potential self-fulfilling prophecy biases. Future studies should aim to systematically report on end-of-life decision-making processes, as this remains a critical factor in interpreting mortality outcomes in this high-risk population. Patients with shock included in the TTM2 trial [30] were not considered in our analysis, as the shock definition used did not clearly distinguish cardiogenic etiology. While this decision limits the scope of the analysis, it was made to preserve pathophysiological consistency and minimize heterogeneity. Unfortunately, a significant portion of the safety outcome analyses is limited by the low number of observed events, leading to wide confidence intervals that encompass the neutral effect and a range of potential effects from benefit to harm. Similarly, the meta-regression results regarding the impact of AMI-CS, temporary MCS, ECMO and cardiac arrest may also have been influenced by the small number of available studies, warranting cautious interpretation.

## Conclusion

In this meta-analysis, therapeutic hypothermia appeared safe but failed to show a significant reduction in all-cause mortality in patients with CS, albeit with a very low certainty of evidence. Given the limited number of studies, small sample sizes, and inconclusive results from trial sequential analyses, it remains premature to draw definitive conclusions regarding either efficacy or inefficacy. Larger, well-designed RCTs are still needed to clarify the role of therapeutic hypothermia in this critically ill population.

## Supplementary Information

Below is the link to the electronic supplementary material.

Supplementary Material 1

Supplementary Material 2

## Data Availability

All data generated or analyzed during this study are included in this published article [and its supplementary information files].

## Abbreviations

AMI: acute myocardial infarction
APACHE: Acute Physiology and Chronic Health Evaluation
CBP: continuous blood purification
CI: cardiac index
CPI: cardiac power index
CS: cardiogenic shock
DO2: oxygen delivery
ECMO: extracorporeal membrane oxygenation
IABP: intra-aortic balloon pump
IRR: incident rate ratio
MCS: mechanical circulatory support
MOF: multiple organ failure
OR: odds ratio
PCWP: Pulmonary capillary wedge pressure
RCT: randomized controlled trial
SBP: systolic blood pressure
SOFA: sequential organ failure assessment
TSA: trial sequential analysis
VO2: oxygen consumption
