# Supplementary material for: Impact of therapeutic hypothermia on cardiogenic shock outcomes: a systematic review and meta-analysis
Source: Ann Intensive Care. 2025 Aug 19;15:122. doi: 10.1186/s13613-025-01541-0 (PMC12364771; doi:10.1186/s13613-025-01541-0)
Supplement: Supplementary file 1 — Supplementary Material 1 [file 13613_2025_1541_MOESM1_ESM.docx]

| **Section and Topic** | **Item #** | **Checklist item** | **Location where item is reported** |
| --- | --- | --- | --- |
| **TITLE** | | |  |
| Title | 1 | Identify the report as a systematic review. | Page 1 |
| **ABSTRACT** | | |  |
| Abstract | 2 | See the PRISMA 2020 for Abstracts checklist. | Page 5 |
| **INTRODUCTION** | | |  |
| Rationale | 3 | Describe the rationale for the review in the context of existing knowledge. | Page 4 |
| Objectives | 4 | Provide an explicit statement of the objective(s) or question(s) the review addresses. | Pages 4 and 5 |
| **METHODS** | | |  |
| Eligibility criteria | 5 | Specify the inclusion and exclusion criteria for the review and how studies were grouped for the syntheses. | Pages 4 and 5 |
| Information sources | 6 | Specify all databases, registers, websites, organisations, reference lists and other sources searched or consulted to identify studies. Specify the date when each source was last searched or consulted. | Page 5  Supplementary Table 2 |
| Search strategy | 7 | Present the full search strategies for all databases, registers and websites, including any filters and limits used. | Pages 4 and 5 |
| Selection process | 8 | Specify the methods used to decide whether a study met the inclusion criteria of the review, including how many reviewers screened each record and each report retrieved, whether they worked independently, and if applicable, details of automation tools used in the process. | Pages 4 and 5 |
| Data collection process | 9 | Specify the methods used to collect data from reports, including how many reviewers collected data from each report, whether they worked independently, any processes for obtaining or confirming data from study investigators, and if applicable, details of automation tools used in the process. | Pages 4 and 5 |
| Data items | 10a | List and define all outcomes for which data were sought. Specify whether all results that were compatible with each outcome domain in each study were sought (e.g. for all measures, time points, analyses), and if not, the methods used to decide which results to collect. | Page 6 |
|  | 10b | List and define all other variables for which data were sought (e.g. participant and intervention characteristics, funding sources). Describe any assumptions made about any missing or unclear information. | Page 6 |
| Study risk of bias assessment | 11 | Specify the methods used to assess risk of bias in the included studies, including details of the tool(s) used, how many reviewers assessed each study and whether they worked independently, and if applicable, details of automation tools used in the process. | Pages 6 and 7 |
| Effect measures | 12 | Specify for each outcome the effect measure(s) (e.g. risk ratio, mean difference) used in the synthesis or presentation of results. | Page 7 |
| Synthesis methods | 13a | Describe the processes used to decide which studies were eligible for each synthesis (e.g. tabulating the study intervention characteristics and comparing against the planned groups for each synthesis (item #5)). | Pages 5 and 6 |
|  | 13b | Describe any methods required to prepare the data for presentation or synthesis, such as handling of missing summary statistics, or data conversions. | Page 7 |
|  | 13c | Describe any methods used to tabulate or visually display results of individual studies and syntheses. | Page 7  Figures 3-5 |
|  | 13d | Describe any methods used to synthesize results and provide a rationale for the choice(s). If meta-analysis was performed, describe the model(s), method(s) to identify the presence and extent of statistical heterogeneity, and software package(s) used. | Page 7  Figures 3-5 |
|  | 13e | Describe any methods used to explore possible causes of heterogeneity among study results (e.g. subgroup analysis, meta-regression). | Pages 8 and 9  Figures 4 and 5 |
|  | 13f | Describe any sensitivity analyses conducted to assess robustness of the synthesized results. | Page 8  Figure 4 |
| Reporting bias assessment | 14 | Describe any methods used to assess risk of bias due to missing results in a synthesis (arising from reporting biases). | Pages 6 and 7  Figure 2 |
| Certainty assessment | 15 | Describe any methods used to assess certainty (or confidence) in the body of evidence for an outcome. | Pages 6 and 7  Supplementary Table 5 |
| **RESULTS** | | |  |
| Study selection | 16a | Describe the results of the search and selection process, from the number of records identified in the search to the number of studies included in the review, ideally using a flow diagram. | Page 9  Figure 1 |
|  | 16b | Cite studies that might appear to meet the inclusion criteria, but which were excluded, and explain why they were excluded. | Figure 1 |
| Study characteristics | 17 | Cite each included study and present its characteristics. | Page 9 Supplementary Table 3 |
| Risk of bias in studies | 18 | Present assessments of risk of bias for each included study. | Figure 2 |
| Results of individual studies | 19 | For all outcomes, present, for each study: (a) summary statistics for each group (where appropriate) and (b) an effect estimate and its precision (e.g. confidence/credible interval), ideally using structured tables or plots. | Figure 3 |
| Results of syntheses | 20a | For each synthesis, briefly summarise the characteristics and risk of bias among contributing studies. | Figure 2 |
|  | 20b | Present results of all statistical syntheses conducted. If meta-analysis was done, present for each the summary estimate and its precision (e.g. confidence/credible interval) and measures of statistical heterogeneity. If comparing groups, describe the direction of the effect. | Pages 10 and 11 Figure 3 |
|  | 20c | Present results of all investigations of possible causes of heterogeneity among study results. | Pages 10 and 11 Figures 4 and 5 |
|  | 20d | Present results of all sensitivity analyses conducted to assess the robustness of the synthesized results. | Pages 10 and 11 Figure 4 |
| Reporting biases | 21 | Present assessments of risk of bias due to missing results (arising from reporting biases) for each synthesis assessed. | Figure 2 |
| Certainty of evidence | 22 | Present assessments of certainty (or confidence) in the body of evidence for each outcome assessed. | Page 11 Supplementary Table 5 |
| **DISCUSSION** | | |  |
| Discussion | 23a | Provide a general interpretation of the results in the context of other evidence. | Pages 11 and 12 |
|  | 23b | Discuss any limitations of the evidence included in the review. | Pages 15 and 16 |
|  | 23c | Discuss any limitations of the review processes used. | Pages 15 and 16 |
|  | 23d | Discuss implications of the results for practice, policy, and future research. | Pages 11-15 |
| **OTHER INFORMATION** | | |  |
| Registration and protocol | 24a | Provide registration information for the review, including register name and registration number, or state that the review was not registered. | Page 5 |
|  | 24b | Indicate where the review protocol can be accessed, or state that a protocol was not prepared. | Page 5 |
|  | 24c | Describe and explain any amendments to information provided at registration or in the protocol. | NA |
| Support | 25 | Describe sources of financial or non-financial support for the review, and the role of the funders or sponsors in the review. | Page 17 |
| Competing interests | 26 | Declare any competing interests of review authors. | Page 17 |
| Availability of data, code and other materials | 27 | Report which of the following are publicly available and where they can be found: template data collection forms; data extracted from included studies; data used for all analyses; analytic code; any other materials used in the review. | Page 17 |

*From:*  Page MJ, McKenzie JE, Bossuyt PM, Boutron I, Hoffmann TC, Mulrow CD, et al. The PRISMA 2020 statement: an updated guideline for reporting systematic reviews. BMJ 2021;372:n71. doi: 10.1136/bmj.n71

**Supplementary Table 1. PRISMA checklist.**

| Database | Search |
| --- | --- |
| MEDLINE via Pubmed | ("hypothermia"[MeSH Terms] OR "hypothermia"[All Fields] OR "hypothermias"[All Fields] OR "hypothermia s"[All Fields] OR ("hypothermia, induced"[MeSH Terms] OR ("hypothermia"[All Fields] AND "induced"[All Fields]) OR "induced hypothermia"[All Fields] OR ("therapeutic"[All Fields] AND "hypothermia"[All Fields]) OR "therapeutic hypothermia"[All Fields]) OR ("mild"[All Fields] AND ("hypothermia"[MeSH Terms] OR "hypothermia"[All Fields] OR "hypothermias"[All Fields] OR "hypothermia s"[All Fields])) OR "sub-hypothermia"[All Fields] OR "Normothermia"[All Fields] OR ("cooled"[All Fields] OR "cooling"[All Fields] OR "coolings"[All Fields] OR "cools"[All Fields]) OR (("moderate"[All Fields] OR "moderated"[All Fields] OR "moderately"[All Fields] OR "moderates"[All Fields] OR "moderating"[All Fields] OR "moderation"[All Fields] OR "moderational"[All Fields] OR "moderations"[All Fields] OR "moderator"[All Fields] OR "moderators"[All Fields]) AND ("hypothermia"[MeSH Terms] OR "hypothermia"[All Fields] OR "hypothermias"[All Fields] OR "hypothermia s"[All Fields]))) AND ("shock, cardiogenic"[MeSH Terms] OR ("shock"[All Fields] AND "cardiogenic"[All Fields]) OR "cardiogenic shock"[All Fields] OR ("cardiogenic"[All Fields] AND "shock"[All Fields]) OR (("acute"[All Fields] OR "acutely"[All Fields] OR "acutes"[All Fields]) AND ("heart failure"[MeSH Terms] OR ("heart"[All Fields] AND "failure"[All Fields]) OR "heart failure"[All Fields])) OR (("acute"[All Fields] OR "acutely"[All Fields] OR "acutes"[All Fields]) AND ("decompensate"[All Fields] OR "decompensated"[All Fields] OR "decompensating"[All Fields] OR "decompensation"[All Fields] OR "decompensations"[All Fields]) AND ("heart failure"[MeSH Terms] OR ("heart"[All Fields] AND "failure"[All Fields]) OR "heart failure"[All Fields]))) |
| EMBASE | ('hypothermia'/exp OR hypothermia OR 'therapeutic hypothermia'/exp OR 'therapeutic hypothermia' OR (therapeutic AND ('hypothermia'/exp OR hypothermia)) OR 'mild hypothermia'/exp OR 'mild hypothermia' OR (mild AND ('hypothermia'/exp OR hypothermia)) OR 'sub hypothermia' OR 'normothermia'/exp OR normothermia OR 'cooling'/exp OR cooling OR 'moderate hypothermia'/exp OR 'moderate hypothermia' OR (moderate AND ('hypothermia'/exp OR hypothermia))) AND ('cardiogenic shock'/exp OR 'cardiogenic shock' OR (cardiogenic AND ('shock'/exp OR shock)) OR 'acute heart failure'/exp OR 'acute heart failure' OR (acute AND ('heart'/exp OR heart) AND ('failure'/exp OR failure)) OR 'acute decompensated heart failure'/exp OR 'acute decompensated heart failure' OR (acute AND decompensated AND ('heart'/exp OR heart) AND ('failure'/exp OR failure))) |
| COCHRANE Library | ("hypothermia"[MeSH Terms] OR "therapeutic hypothermia"[TW] OR "mild hypothermia"[TW] OR "sub hypothermia"[TW] OR "normothermia"[TW] OR "cooling"[TW] OR "moderate hypothermia"[TW]) AND ("cardiogenic shock"[MeSH Terms] OR "acute heart failure"[TW] OR " acute decompensated heart failure "[MeSH Terms]) |
| SCOPUS | (TITLE-ABS-KEY (hypothermia OR (therapeutic AND hypothermia) OR (mild AND hypothermia) OR (sub AND hypothermia) OR normothermia OR (moderate AND hypothermia))) AND (TITLE-ABS-KEY ((cardiogenic AND shock) OR (acute AND heart AND failure) OR (acute AND decompensated AND heart AND failure))) |
| Web of Science | ALL=((hypothermia OR therapeutic hypothermia OR mild hypothermia OR sub hypothermia OR normothermia OR moderate hypothermia) AND (cardiogenic shock OR acute heart failure OR acute decompensated heart failure)) |

**Supplementary Table 2. Details of MeSH Search Terms**

Titles and abstracts of studies retrieved using the search strategy were independently screened by 2 investigators (C. D. and M. C.) to identify studies with an active arm involving therapeutic hypothermia in patients hospitalized with CS. No minimum sample size was required for inclusion. The full texts of potentially eligible studies were retrieved and independently assessed for eligibility by the two investigators. Discrepancies between investigators were discussed collegially and the final decision was then made by consensus.

|  | **Zobel et al.** | **Orban et al.** | **Blatt et al.** | **SHOCK-COOL** | **Fang et al.** | **HYPO-ECMO** | **CHILL-SHOCK** |
| --- | --- | --- | --- | --- | --- | --- | --- |
| **Trial N^o^ identifier** | - | - | - | NCT01890317 | - | NCT02754193 | NCT03141255 |
| **Country** | Germany | Germany | Israel | Germany | China | France | USA |
| **Study design** | Prospective, propensity-score matched, two-arms, open-label, single center | Prospective, two-arms, open-label, multicenter | Prospective, two-arms, open-label, single center | RCT, two-arms, open-label, single-center | Prospective, randomized, two-arms, open-label, single center | RCT, two-arms, open-label, multicenter | RCT, two-arms, open-label, single-center |
| **Main inclusion criteria** | - Out-of-hospital cardiac arrest  - Cardiogenic shock: clinical criteria (hypotension SBP <90 mm Hg for > 30 mins or the need for supportive measures to maintain a SBP > 90 mm Hg, end-organ hypoperfusion  [cool extremities]), hemodynamic criteria (cardiac index < 2.2  L/min/m²)  - Glasgow Coma Score <8 | - AMIC-CS  - Cardiogenic shock: hypotension SBP < 90 mm Hg for >  30 minutes or the need for pressure support with catecholamines to  maintain SBP ≥ 90 mm Hg), clinical signs of pulmonary congestion and impaired end-organ perfusion (altered mental status; cold, clammy skin and extremities; oliguria with urine output < 30 ml/h; or serum lactate > 2.0 mmol/l | - AMIC-CS (anterior or inferior STEMI)  - Age 18-75 years  - Duration of symptoms < 12 h  - Persistent hypotension, systolic pressure < 90 mm Hg despite fluids and catecholamine infusion | - AMI-CS  - SBP <90 mm Hg for >30 min or catecholamines required to maintain a SBP >90 mm Hg  - Absence of hypovolemia  - Signs of pulmonary congestion  - Signs of impaired organ perfusion defined by at least 1 of the following: altered mental status; cold, clammy skin; urine output <30 mL/h; or arterial lactate >2 mmol/L.  - Intubation, sedation, and invasive ventilation | - - Postoperative CS: blood pressure < 90/60 mmHg and cardiac index <2.5 L/min.m^2^ | - AMI and non-AMI CS  - Age ≥ 18 years  - Intubated patients with CS treated with VA-ECMO  - Patient affiliated to social security plan | - AMI (STEMI, NSTEMI) and non-AMI (myocarditis, HCM, stress CM, peripartum CM, CS with preserved ejection fraction) CS  - Age ≥ 18 years AND ≤ 89 years  - SBP <90mmHg for ≥ 30’, CI < 2.2 L/min/m2, PCWP ≥ 15mmHg, need for central venous access, vasopressors, inotropes and/or to maintain SBP ≥ 90mmHg |
| **Main exclusion criteria** | NA | - No available  platelet function test  - Concomitant therapy with a glycoprotein IIb/IIIa inhibitor  - No administration of a loading dose of an ADP-receptor  antagonist due to a higher bleeding risk | - Cardiac arrest  - Known congestive heart failure  - Significant valve disease  - CS related to any mechanical complication (rupture free wall, ventricular septal defect, tamponade, acute mitral regurgitation, significant right ventricle infarction)  - End-stage kidney disease or hepatic failure  - Recent stroke | - CS duration >12 hours  - Cardiopulmonary resuscitation with an indication for temperature control according to current guidelines | - Pre-operative coronary atherosclerotic heart disease or peri-operative myocardial ischemia  - Secondary surgical trauma | - Out of hospital refractory cardiac arrest  - Implantation of VA ECMO under cardiac massage with a duration of cardiac massage >45minutes  - VA-ECMO after cardiac surgery for heart transplantation or lung transplantation or left or biventricular assist device implantation  - VA-ECMO for acute poisoning with cardio-toxic drugs  - Uncontrolled bleeding  - Cerebral deficit with fixed dilated pupils | - Recent cardiotomy  - History of cardiac transplantation  - Baseline heart rate < 60 bpm  - Baseline temperatures < 35°C |
| **Hypothermia targeted temperature** | - Targeted temperature 33°C applied for 24h | - Targeted temperature 32 to 34°C applied for  24 hours | - Targeted temperature 32 to 34°C applied for 12 hours | - Target temperature of 33°C | - Targeted temperature 34.0°C to 35.0°C applied for 24 hours | - Target temperature of 33 °C to 34 °C for 24 hours | - Target temperature 32°C - 34°C for 24 hours |
| **Hypothermia protocol** | - Rapid infusion of 2 L of ice cold saline (4°C) by the emergency team  - Endovascular  cooling device (CoolGard 3000/ ICY catheter, Zoll Medical, Chelmsford, MA)  - Core temperature continuously registered in the bladder through a thermal sensor at the tip of a transurethral urinary catheter | - Non-invasive Arctic Sun® Temperature Management system (Medivance, Inc.,  Louisville, Colorado) started as soon as possible after arrival of the patient on the ICU  - Temperature control with the manufacturer´s computer algorithm and secondary  esophageal or bladder temperature sensors | - Non-invasive induction by combining an IV infusion of normal saline at 4°C alongside to a designated hypothermia suit  - After induction, hypothermia  was maintained by the suit only  - Body temperature was monitored independently using  3 separate probes. | - Cooling maintained with a commercially available system (CoolGard, ZOLL Medical Corp) for 24 hours with central temperature measurement in the urinary bladder  - After the target temperature was reached, it was maintained for 24 hours with the automatic temperature regulation function of the CoolGard system by central temperature measurement in the  urinary bladder | - Blood purification carried out at the bedside  - Continuous veno-venous blood filtration mode with an improved Port formula, pre-diluted, no anticoagulant | - Adjustment of the venoarterial ECMO circuit temperature controller  - Core temperature had to be monitored through an esoph-  ageal or bladder probe | - Cooling with the Thermogard XP temperature  management system (ZOLL), along with the Quattro cooling catheter  - Core temperature was measured via a  thermal tip at the end of a urinary catheter or an endotracheal temperature probe |
| **Target temperature achievement** | Target temperature successfully reached in all patients  Mean time to reach target temperature: 79 ± 14 minutes | Temperature level was lowered  insufficiently in 6 patients (9%) to 35 °C and in 3 patients (5%)  to 36 °C | Target temperature successfully reached in all patients  Mean time to reach target temperature: 118 ± 27 minutes | Target temperature successfully reached in all patients | Not specified | Target temperature successfully reached in all patients | Not specified |
| **Rewarming** | Rewarming through endovascular cooling device at a controlled rate of 0.3°C per hour until reaching 37°C | Rewarming was started 24h after resuscitation elevating temperature automatically by the Arctic Sun® Temperature  Management system | Re-warming was initiated after  12 h of hypothermia at a rate of 0.3°C per hour | Rewarming with a speed of 0.25°C/h to 37.0°C | Not specified | Rewarming with a speed of 0.1-  0.2 °C per hour to 37 °C | Rewarming with a speed of 0.3°C per hour to 37°C. |
| **Primary endpoints** | Hierarchy of endpoints not specified | Hierarchy of endpoints not specified | 30-day all-cause mortality | CPI at 24 hours after randomization | CI  DO_2_/VO_2_ ratio  APACHE III score  MODS score | 30-day mortality | Composite of: bradycardia requiring medical therapy or a temporary pacemaker, bleeding requiring transfusions, uncontrolled shivering, hyperglycemia requiring insulin therapy in a patient without known diabetes mellitus, electrolyte derangements unexplained by underlying medical conditions, and other complications related to the insertion of the cooling catheter |
| **Main secondary endpoints** | Hierarchy of endpoints not specified | Hierarchy of endpoints not specified | - AMI size, assessed by cardiac markers (troponin T and creatinine kinase)  - TH-related complications: infection, refractory arrhythmias | - Mean arterial and pulmonary arterial blood pressures  - Arterial lactate measured every 2 hours  - Duration of mechanical ventilation and ICU stay  - Acute Physiology II score  - Sepsis, pneumonia, and bleeding | - Bradycardia  - Ventricular arrhythmia  - Muscle tremors  - ICU duration  - Mechanical ventilation time  - CBP time  - Infection rate  - Blood loss  - Mortality | - Mortality at 48 hours and at days 7, 60, and 180  - VA-ECMO duration  Composite outcome of death, heart transplant, escalation to LVAD implantation  - Length of use of vasopressors in the ICU  - Time to normalization of lactate level  - Duration of mechanical ventilation, number of ventilator-free days at 30, 60, and 180 days  - Kidney replacement therapy–free days at days 30, 60, and 180  - Duration of ICU and hospital stay  - Bleeding  - Sepsis (pulmonary, bloodstream, cannula-related ECMO)  - Number of units of packed red blood cells transfused during VA ECMO | - 90-day all-cause mortality  - Hemodynamic parameters  - Echocardiographic parameters  - Cumulative vasopressor and inotrope dosage requirements |
| **Last follow-up** | Not specified | 30 days | 30 days | 2 years | 3 days | 180 days | 90 days |

**Supplementary Table 3. Study Protocols of Randomized Trials Included in the Meta-Analysis.**

AMI, acute myocardial infarction; AMI-CS, acute myocardial infarction related cardiogenic shock; APACHE, Acute Physiology and Chronic Health Evaluation; CBP, continuous blood purification; CI, cardiac index; CM, cardiomyopathy; CPI, cardiac power index; CS, cardiogenic shock; DO_2_, oxygen delivery; HCM, hypertrophic cardiomyopathy; HF, heart failure; ICU, intensive care unit; LVAD, left ventricular assist device; MCS, mechanical circulatory support; MI, myocardial infarction; MODS, multiple organ dysfunction syndrome; NSTEMI, non-ST-elevation myocardial infarction; PCWP, Pulmonary capillary wedge pressure; RCT, randomised controlled trial; SBP, systolic blood pressure; SOFA, sequential organ failure assessment; STEMI, ST-elevation myocardial infarction; TH, therapeutic hypothermia; VA-ECMO, venoarterial extracorporeal membrane oxygenation; VO_2_, oxygen consumption

|  | Outcome | Odds ratio | 95% CI | p value |
| --- | --- | --- | --- | --- |
| AMI-CS | All-cause mortality (longest follow-up) | 1.70 | 0.77 – 3.75 | 0.19 |
|  | 30-day all-cause mortality | 1.83 | 1.01 – 3.35 | 0.05 |
|  | Bleeding | 3.57 | 2.55 – 5.00 | < 0.01 |
| MCS (Impella or IABP or ECMO) | All-cause mortality (longest follow-up) | 1.13 | 0.62 – 2.07 | 0.70 |
|  | 30-day all-cause mortality | 0.48 | 0.44 – 0.51 | < 0.01 |
|  | Bleeding | 0.54 | 0.37 – 0.77 | < 0.01 |
| ECMO | All-cause mortality (longest follow-up) | 1.14 | 0.64 – 2.01 | 0.67 |
|  | 30-day all-cause mortality | 0.48 | 0.44 – 0.51 | < 0.01 |
|  | Bleeding | 0.60 | 0.33 – 1.08 | 0.09 |
| Cardiac arrest | All-cause mortality (longest follow-up) | 0.43 | 0.17 – 1.13 | 0.09 |
|  | 30-day all-cause mortality | 0.72 | 0.31 – 1.27 | 0.20 |

**Supplementary Table 4. Meta regressions for efficacy and safety outcomes according to AMI-CS and MCS**

AMI-CS, acute myocardial infarction related cardiogenic shock; CI, confidence interval; ECMO, extracorporeal membrane oxygenation; IABP, intra-aortic balloon pump; MCS, mechanical circulatory support; SE, standard error

| Participants | Risk of bias | Inconsistency | Indirectness | Imprecision | Publication bias | Overall certainty of evidence |
| --- | --- | --- | --- | --- | --- | --- |
|  |  |  |  |  |  |  |
| **30-day all-cause mortality** | | | | | | |
| 540 patients  2 RCTs  2 case-control studies | Very serious^a^ | Not serious | Not serious | Serious^b^ | Not serious | ⨁◯◯◯ Very low |
| **All-cause mortality** | | | | | | |
| 695 patients  4 RCTs  3 case-control studies | Very serious^a^ | Not serious | Not serious | Serious^b^ | Not serious | ⨁◯◯◯ Very low |
| **Pneumonia** | | | | | | |
| 374 patients  2 RCTs | Not serious | Not serious | Not serious | Serious^b^ | Not serious | ⨁⨁◯◯ Low |
| **Sepsis** | | | | | | |
| 374 patients  2 RCTs | Not serious | Not serious | Not serious | Serious^b^ | Not serious | ⨁⨁◯◯ Low |
| **Bleeding** | | | | | | |
| 539 patients  3 RCTs  1 case-control study | Very serious^a^ | Not serious | Not serious | Serious^b^ | Not serious | ⨁◯◯◯ Very low |

**Supplementary Table 5. GRADE evidence profile**

^a^Due to the inclusion of non-randomized studies

^b^Due to the inclusion of studies with small sample sizes

CI, confidence interval; RCT, randomized controlled trial


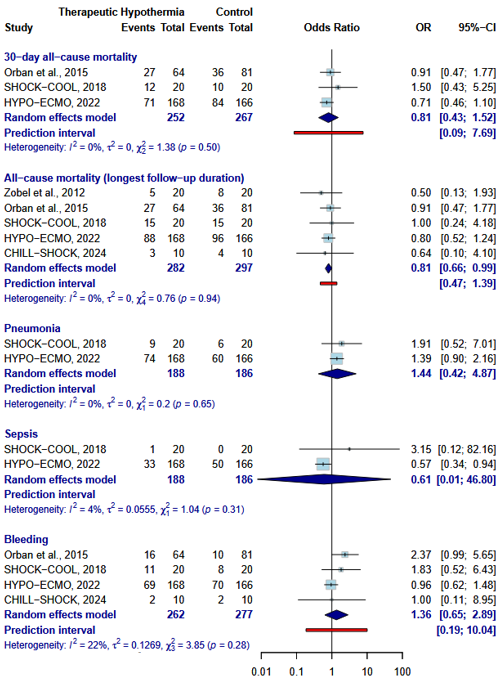


Supplementary Figure 1. Sensitivity analysis of efficacy and safety endpoints for therapeutic hypothermia in cardiogenic shock patients, including only studies with a target temperature of 32–34 °C applied for 24 hours.

CI, confidence interval; OR, odds ratio


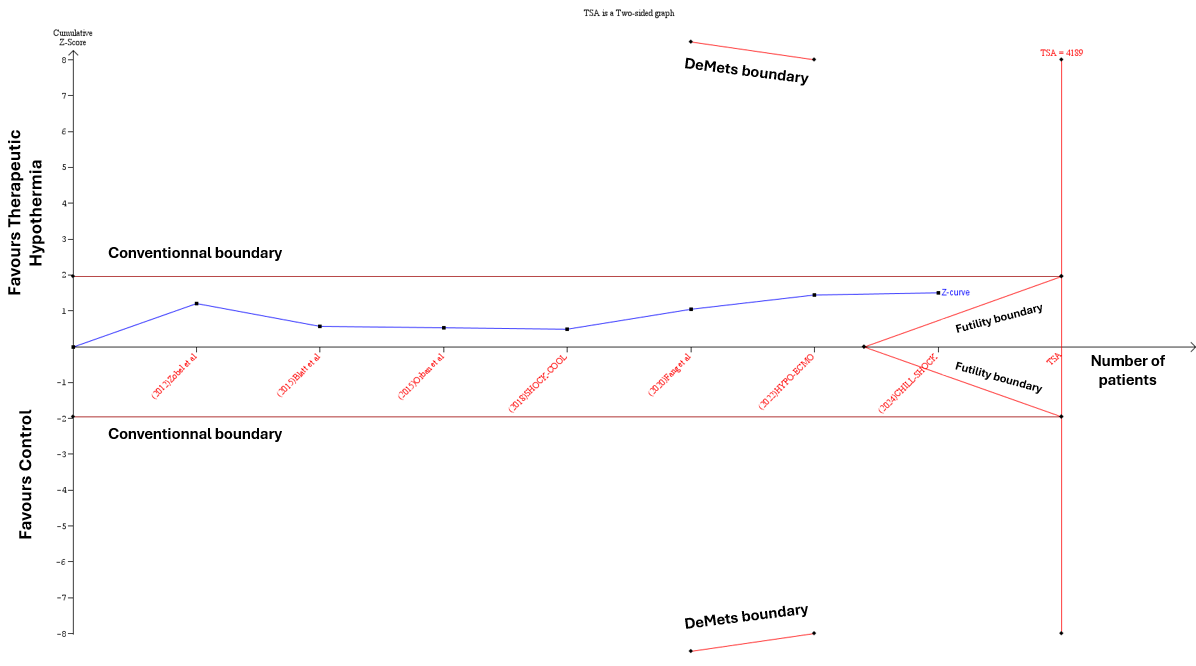


Supplementary Figure 2. Trial sequential analysis of all-cause mortality outcome at the longest follow-up duration.

The blue Z-curve represents the cumulative evidence from included studies plotted against the cumulative sample size. The horizontal red lines represent conventional boundaries for statistical significance (α = 0.05), while the slanted red lines show the trial sequential monitoring boundaries (DeMets boundaries) and futility boundaries. The required information size was calculated as 4,356 patients assuming a 10% relative risk reduction, 5% type I error, and 90% power.
