# Supplementary material for: Impact of therapeutic hypothermia on cardiogenic shock outcomes: a systematic review and meta-analysis
Source: Ann Intensive Care. 2025 Aug 19;15:122. doi: 10.1186/s13613-025-01541-0 (PMC12364771; doi:10.1186/s13613-025-01541-0)
Supplement: Supplementary file 2 — Supplementary Material 2 [file 13613_2025_1541_MOESM2_ESM.docx]

First, we would like to thank the Editors and the Reviewers for the chance to submit a revised version of our manuscript. Thank you very much for taking the time to review our manuscript and for providing us with your valuable feedback. Please find below our responses to each comment in red and corresponding changes in the manuscript.

Editors' comments (if any):
This is an interesting set of data. However, as mentioned by one of the reviewers, the primary outcome registered in PROSPERO is 30-day mortality, not long term mortality. While the DangerShock trial has illutrated the interest of long term outcome, this may have been anticipated at trial registration on Jan 2025.
Hence the manuscript should be reorganized, with 30 days  as main outcome as decided originally at time of study registration, and the other outcomes should be considered as secondary outcomes.

We thank the Editor for his appreciation and for this important comment, as well as for the opportunity to clarify our rationale regarding the selection of the primary outcome.

As correctly noted, our PROSPERO registration listed 30-day all-cause mortality as the primary outcome. However, following extensive discussions within our scientific committee, we initially chose to prioritize all-cause mortality at the longest follow-up duration in the manuscript. This decision was based on three main considerations:

- Indeed, as mentioned, studies such as Danger-Shock have demonstrated that early interventions in CS, even including short-duration treatments, may lead to delayed benefits through improved myocardial recovery, thereby influencing long-term outcomes
- From a methodological standpoint, more data were available at the longest follow-up, which was deemed particularly relevant given the limited number of studies in the field of hypothermia in CS
- Other large-scale, authoritative studies referenced in hypothermia guidelines, such as the TTM2 trial, have also evaluated medium- to long-term mortality, typically at 6 months

That said, we fully acknowledge the clinical relevance of 30-day mortality, especially in the acute setting of CS, and agree that it should be prioritized in line with our original study registration.

Accordingly, we have substantially revised the manuscript to designate 30-day all-cause mortality as the primary outcome, and all-cause mortality at the longest follow-up as a secondary outcome. Below are the key changes implemented in the revised manuscript:

- Abstract – Results: “*Hypothermia was associated with improved survival at the longest available follow-up (OR 0.79 [0.62–0.99]), but not at 30 days (OR 0.83 [0.54–1.26]). Sensitivity analysis adjusted for follow-up duration confirmed a trend toward reduced all-cause mortality (IRR 0.85 [0.72–1.01]).*” → replaced with: “*Hypothermia was not associated with a significant reduction in all-cause mortality at 30 days (OR 0.83 [0.54–1.26]) or at the longest available follow-up (IRR 0.85 [0.72–1.01]).*”
- Abstract – Conclusion: “*In this meta-analysis, therapeutic hypothermia appeared to be safe and was associated with a potential reduction in all-cause mortality in patients with CS, albeit with very low level of evidence*.” → replaced with: “*In this meta-analysis, therapeutic hypothermia appeared safe but failed to show a significant reduction in all-cause mortality in patients with CS, albeit with very low certainty of evidence. Larger RCTs are warranted to clarify its clinical utility*.”
- Outcomes Section: “*The primary outcome was all-cause mortality. Secondary outcomes included rates of pneumonia, sepsis, bleeding, and supraventricular or ventricular arrhythmias. Each study's definitions of these events were used in the analysis, and events reported at the longest follow-up time point were extracted*.” → replaced with: “*The primary outcome was 30-day all-cause mortality. Secondary outcomes included all-cause mortality at the longest reported follow-up, as well as the incidence of pneumonia, sepsis, bleeding, and supraventricular or ventricular arrhythmias. For each outcome, the definitions provided in the original studies were used in the analysis*.”
- TSA Methods: we clarified: “*The TSA was conducted for the primary outcome of 30-day all-cause mortality and for all-cause mortality at the longest reported follow-up to assess the robustness of our findings*.”
- Results Section: The paragraph starting with “*Figure 3A shows that therapeutic hypothermia was associated with a significant reduction*…” → replaced with: “*Figure 3A shows that therapeutic hypothermia was not associated with a significant reduction in all-cause mortality at 30 days (OR 0.83 [0.54–1.26], I² = 0%, τ² = 0, PI 0.39–1.75). At the longest follow-up, a modest reduction was observed (OR 0.79 [0.62–0.99]), with low heterogeneity, though the PI included both benefit and neutrality (0.52–1.19). Conversely, sensitivity analysis adjusted for follow-up duration revealed the absence of a statistically significant reduction (IRR 0.85 [0.72–1.01]) (Figure 3B).*”
- TSA Results Section: Previously: “*TSA for the primary outcome of all-cause mortality showed a trend toward improvement…*” → replaced with: “*TSA for the primary outcome of 30-day all-cause mortality showed no statistically significant difference between therapeutic hypothermia and control groups, as the Z-curve did not cross the conventional boundary (Figure 5). The Z-curve also did not cross the futility boundary or reach the required information size (n = 4356), indicating that the current evidence is underpowered to detect or exclude a clinically relevant difference. Similarly, TSA for all-cause mortality at the longest follow-up confirmed the absence of a statistically significant benefit, with the Z-curve crossing neither the conventional nor futility boundaries and not reaching the required information size (Supplementary Figure 2). Both analyses indicate that current evidence is insufficient to demonstrate a benefit of therapeutic hypothermia in CS patients, and substantially more data would be needed to draw definitive conclusions*.”
- Main Discussion Points: point 1 and point 4 revised as follows: *1) Based on currently available data, therapeutic hypothermia is not associated with a statistically significant reduction in all-cause mortality in CS, although the certainty of evidence is very low*; *4) Despite a strong physiological rationale and encouraging preclinical data, prospective clinical studies remain scarce, and further research is still warranted to clarify the potential role of hypothermia in this setting*.
- Discussion – Interpretation: Previously: “*our main analysis found that therapeutic hypothermia was associated with a significant 21% reduction…*” → replaced with: “*In our main analysis, therapeutic hypothermia failed to show a significant reduction in 30-day all-cause mortality. Similarly, no improvement in overall survival was observed at the longest follow-up in sensitivity analyses or after adjustment using incidence rate ratios*.”
- Discussion – Limitations: “*The lack of robustness in the observed benefit*…” → replaced with: “*The lack of observed survival benefit may also result from insufficient statistical power, due to the limited number of studies and small sample sizes*.”
- Discussion – General Interpretation: “*Our study illustrates that although hypothermia showed encouraging results in pre-clinical CS studies*…” → replaced with: “*Overall, our study illustrates that, although hypothermia has shown encouraging results in preclinical CS studies, there is currently insufficient data to support its effectiveness in clinical practice. However, at this stage of research, it is still too early to formally conclude its inefficacy, given the limited number of studies conducted, as highlighted by the results of the trial sequential analysis*”
- Conclusion (Full Text): “*In this meta-analysis, therapeutic hypothermia appeared safe but failed to show a significant reduction in all-cause mortality in patients with CS, albeit with a very low certainty of evidence. Given the limited number of studies, small sample sizes, and inconclusive TSA results, it remains premature to draw definitive conclusions regarding either efficacy or inefficacy. Larger, well-designed RCTs are still needed to clarify the role of therapeutic hypothermia in this critically ill population*.”
- Figures and Supplementary Materials: We reorganized *Figures 3* and *4* and *Supplementary Table 5* to reflect 30-day all-cause mortality as the primary outcome. We added *Figure 5* for the TSA on 30-day mortality and moved the TSA on longest follow-up to *Supplementary Figure 2*. We chose to retain both TSA analyses, as the longest follow-up includes more patients and could have yielded different insights. However, the similar findings between the two strengthen the consistency and robustness of our results.

In conclusion, we hope these substantial revisions clarify our interpretation and reinforce our commitment to methodological rigor. Despite encouraging preclinical data, therapeutic hypothermia failed to demonstrate a clinical benefit in CS based on currently available data. Nevertheless, our study highlights a critical knowledge gap and provides a strong rationale for the conduct of larger-scale clinical trials in this area.

Reviewer #1: Dear Editor, thank you for the opportunity to review the manuscript by Cherbi et al about the impact of therapeutic hypothermia on cardiogenic shock outcomes. The Authors have conducted a systematic review and a meta-analysis, suggesting that the current evidence remains inconclusive. The study is well written, and the methods used are in line with the good practice reports (PROSPERO, PRISMA, ROB2, ROBINS-I, GRADE).
The topic is of interest, but I have some elements that I would like to discuss.

First of all, we would like to thank the reviewer for his/her numerous and very insightful comments, which have helped us to deepen several important aspects of our manuscript.
1) as correctly reported in the PROSPERO file, the element for PICO was an exposure to TH in the range of 33-34°C for 24h, yet "all therapeutic hypothermia protocols were included" is reported in the eligibility criteria. As longer duration of the treatment, different degree of hypothermia as well as fever control may have different impact, a clarification is needed, since one study was included that assessed a different target, and another for less than 24h.
We thank the reviewer for highlighting this important methodological point and for giving us the opportunity to provide clarification. Indeed, this issue was the subject of extensive discussion within our scientific committee.

During the initial systematic search, it quickly became apparent that only a limited number of studies had investigated therapeutic hypothermia in the setting of cardiogenic shock. Faced with this restricted body of literature and in an effort to maximize the statistical power of our analysis, we made the decision to broaden our inclusion criteria to encompass:

- Hypothermia protocols targeting slightly different temperatures (e.g., 34–35°C instead of the pre-specified 32–34°C),
- Treatment durations shorter than 24 hours.

We fully acknowledge that this may introduce a degree of heterogeneity into the analysis and interpretation of our results. However, after careful deliberation, we considered that the potential impact of this broader selection would likely be limited, for the following reasons:

- Of the 7 studies included, only one (Fang et al., n = 95/695 patients) used a hypothermia protocol with a target temperature outside the 32–34°C range, specifically 34–35°C.
- Similarly, only one study (Blatt et al., n = 21/695 patients) implemented a hypothermia protocol of a shorter duration (12 hours instead of 24). Details of the hypothermia regimens applied are now specifically provided in *Supplementary Table 3*.
- Several preclinical studies have suggested that while hypothermia induces substantial physiological changes compared to normothermia, significant hemodynamic differences between varying degrees of hypothermia are not consistently observed (PMID: 11327339, PMID: 3383383; referenced as studies 27 and 28 in the manuscript).

Overall, the degree of heterogeneity observed in our meta-analysis remained low across all outcomes, regardless of the index used (I², prediction interval, τ²), even when including the studies by Fang et al. and Blatt et al.

That said, we fully understand and appreciate the reviewer’s concern. To address it, we performed a sensitivity analysis restricted to the five studies applying a standardized therapeutic hypothermia protocol with a target temperature of 32–34°C maintained for 24 hours (i.e., excluding Blatt et al. and Fang et al.). Overall, results were consistent with those of the main analysis.

Accordingly, and to ensure these elements are transparently reflected, we modified the manuscript as follows:

- In the "Sensitivity analyses" section of the Methods, we clarified: *“**Additionally, three other sensitivity analyses were performed: (1) excluding the HYPO-ECMO trial, which included the majority of patients, to assess the robustness of the results; (2) restricting the analysis to studies with a low risk of bias; (3) restricting the analysis to studies applying a therapeutic hypothermia protocol with a target temperature of 32–34°C maintained for 24 hours.”*
- We added a new figure reporting the results of this analysis as *Supplementary Figure 1*.
- In the Results section, we added: *“**Finally, the sensitivity analysis restricted to studies applying hypothermia between 32–34°C for 24 hours also yielded results consistent with the main analysis (Supplementary Figure 1).”*
- In the Discussion, we clarified: *“There was some heterogeneity in the application of therapeutic hypothermia across studies, including differences in the timing of initiation, cooling methods (external surface devices, intravascular catheters, ECMO circuit adjustment, or cold fluid infusion), and duration. While most studies used controlled systems with continuous core temperature monitoring and feedback-regulated devices, the methods varied across trials. Hypothermia was maintained for 24 hours in six studies, while one study (Blatt et al.) applied it for only 12 hours. Only one study (Fang et al.) targeted a temperature range outside of 32–34°C, aiming instead for 34–35°C. Data on the exact time to reach the target temperature and on rewarming protocols were inconsistently reported, which limits the ability to fully assess the impact of cooling and rewarming rates on outcomes. Nevertheless, results from the sensitivity analysis restricted to studies applying a 24-hour hypothermia protocol with a 32–34°C target were consistent with the main analysis. Moreover, preclinical studies (27, 28) suggest that although hypothermia induces significant physiological changes compared to normothermia, hemodynamic differences between various hypothermic targets are not consistently observed.”*
  2) no information about the technique to induce and maintain hypothermia is provided (surface? intravascular? cold fluids? feedback system or blanket?): this is important, as in some trials, the effect may have been dependent on the technique used.
  I would also report the rate of target temperature achievement in each study, because if low quality TH was implemented, the results may differ from high quality TH.
  3) as much as the cooling rate, the rewarming rate might be important.

We thank the reviewer for highlighting these critical methodological aspects. Detailed information regarding the hypothermia protocol has been added in *Supplementary Table 3*. To enhance clarity and directly address the reviewer’s concerns, we created four dedicated sections within the table: *“Hypothermia targeted temperature”*, *“Hypothermia protocol”*, *“Target temperature achievement”*, and *“Rewarming”*, to provide a more granular description of these key elements.

The techniques used to induce and maintain hypothermia included:

- Endovascular cooling systems (e.g., CoolGard®/ICY® catheter) in three studies,
- Non-invasive surface cooling systems (e.g., Arctic Sun® or hypothermia suit) in two studies,
- Intravenous infusion of cold fluids (4 °C) for rapid induction in at least three studies,
- ECMO circuit temperature modulation or continuous veno-venous hemofiltration in two studies.

Additionally, all studies employed continuous core temperature monitoring via bladder, esophageal, or multi-site probes, except for Fang et al., in which the monitoring method was not specified.

Regarding target temperature achievement:

- Four out of seven studies reported a 100% success rate among included patients,
- Orban et al. reported insufficient temperature reduction in 6 patients (9%) to 35 °C and in 3 patients (5%) to 36 °C,
- In two studies—Fang et al. (n = 95/695 patients) and CHILL-SHOCK (n = 20/695 patients)—the exact achievement rate was not reported.

Rewarming was performed in a relatively homogeneous way across studies, with a rate ranging from 0.1 to 0.3 °C per hour until reaching 37.0 °C, except in Fang et al., where the rewarming rate was not specified.

As a result, we have implemented the following important modifications to the manuscript:

- First, the studies by Fang et al. and CHILL-SHOCK lacked sufficient details on at least one aspect of the hypothermia and rewarming protocol. Consequently, after team discussion, we downgraded the risk of bias for both studies to “Some concerns,” in accordance with RoB2 Domain 2 criteria (deviations from intended interventions, with “No information” available). A revised sensitivity analysis including only studies at low risk of bias was performed and did not affect the overall findings; *Figure 4B* and the main text have been updated accordingly.
- Overall, although a 100% success rate in target temperature achievement was not reached across all studies, it was achieved in 571 out of 580 patients for whom this information was reported—excluding Fang et al. and CHILL-SHOCK. This represents a very low protocol deviation rate, consistent with what can be expected in intention-to-treat analyses.
- In the Results section: “*All interventional groups targeted a temperature range of 32 to 34°C, except for the study by Fang et al. (11), which aimed for a target of 34 to 35°C. In six out of seven studies, the targeted temperature was maintained for 24 hours, except for the study by Blatt et al. (9), where the duration was 12 hours. Nearly all patients in the therapeutic hypothermia groups achieved the targeted temperature (571 out of 580 patients; data were not reported for the remaining 115).”*
- In the Discussion section: “*There was some heterogeneity in the application of therapeutic hypothermia across studies, including differences in the timing of initiation, cooling methods (external surface devices, intravascular catheters, ECMO circuit adjustment, or cold fluid infusion), and duration. While most studies used controlled systems with continuous core temperature monitoring and feedback-regulated devices, the methods varied across trials. Hypothermia was maintained for 24 hours in six studies, while one study (Blatt et al.) applied it for only 12 hours. Only one study (Fang et al.) targeted a temperature range outside of 32–34°C, aiming instead for 34–35°C. Data on the exact time to reach the target temperature and rewarming protocols were inconsistently reported, which limits the ability to fully assess the impact of cooling and rewarming rates on outcomes. Nevertheless, results from the sensitivity analysis restricted to studies applying a 24-hour hypothermia protocol with a 32–34°C target were consistent with the main analysis. Moreover, preclinical studies (27, 28) suggest that although hypothermia induces significant physiological changes compared to normothermia, hemodynamic differences between various hypothermic targets are not consistently observed*.”

4) the results are dampened by the important number of patients with a cardiac arrest (49% of the total): in this cohort of patients, we know that the intervention is not really effective in reducing mortality, even when patients are in shock. Furthermore, the mortality is often driven by neurological reasons, and protocolized WLST is fundamental in this context to minimize the biases of self-fulfilling prophecy, hence the results on mortality are hard to interpret without knowing how these studies defined this specific decision. This is even more important since the mortality in the populations of AMI-CS was higher in the TH group.
I also consider hard not to define in cardiogenic shock the patients in which shock was reported in the TTM trials, which were not included in the analysis (482 in total only in the TTM2). A specific comment to justify why those were not included while the selected study did.

First, we acknowledge that none of the studies included in this meta-analysis—clinical trials included—provided comprehensive information on ethical protocols or the management of WLST. This clearly represents an important limitation. On the other hand, this may also be seen as a reflection of real-life practices, where center-specific approaches and considerable heterogeneity exist, especially in such a complex and nuanced area as end-of-life decisions. Finally, although this does not constitute formal evidence, it is worth noting that baseline characteristics such as age or comorbidities—factors that often guide WLST decisions—were not markedly different between the normothermia and hypothermia groups. This point was addressed in the 'Limitations' section as follows: “*None of the included studies reported detailed information on withdrawal of life-sustaining therapy protocols, which limits the ability to account for potential self-fulfilling prophecy biases. Future studies should aim to systematically report on end-of-life decision-making processes, as this remains a critical factor in interpreting mortality outcomes in this high-risk population.”*

We particularly thank the reviewer for his/her comment regarding the TTM2 trial, as this point was extensively discussed within our team.

After a detailed analysis, we ultimately chose not to include this study for several reasons:

- First, the TTM2 trial (PMID: 34133859) included approximately 30% of patients with “shock,” defined by the authors as “systolic blood pressure of less than 90 mm Hg for more than 30 minutes or end-organ hypoperfusion (cool arms and legs, urine output <30 ml per hour, and heart rate <60 beats per minute).” Our study specifically aimed to evaluate therapeutic hypothermia in cardiogenic shock. Unfortunately, the definition of shock in TTM2 does not clearly establish cardiogenic origin. While it is likely that a proportion of these patients did have cardiogenic or mixed shock, we considered it more appropriate not to include them to avoid introducing significant heterogeneity—particularly from patients with septic shock (in whom temperature control is inherently more difficult) or hemorrhagic shock (for which the rationale for hypothermia would differ fundamentally from that in cardiogenic shock).
- Moreover, the TTM2 authors have already addressed this issue in their original publication, which includes a subgroup analysis based on shock status showing no survival benefit of hypothermia (RR 1.01 [0.89–1.15]). Additionally, a separate meta-analysis focusing on patients with shock from both TTM and TTM2 concluded that hypothermia at 33°C did not decrease 6-month mortality compared with normothermia after out-of-hospital cardiac arrest (PMID: 38319850).

To justify our choice, we have added the following statement to the Limitations section:
"*Patients with shock included in the TTM2 trial were not considered in our analysis, as the shock definition used did not clearly distinguish cardiogenic etiology. While this decision limits the scope of the analysis, it was made to preserve pathophysiological consistency and minimize heterogeneity.*"

Lastly, in order to address the reviewer’s concern as thoroughly as possible despite the limited available data, we conducted an additional meta-regression analysis to assess the influence of the proportion of cardiac arrest patients on all-cause mortality at both 30 days and the longest follow-up duration.

Accordingly:

- The Methods section was updated to include the following: " *Eventually, in light of gaps in the evidence and previous studies (1, 7–13), a meta-regression analysis was conducted to assess the effect of AMI-CS, temporary MCS (Impella or intra-aortic balloon pump [IABP] or extracorporeal membrane oxygenation [ECMO]), and cardiac arrest on mortality*."
- The results of this analysis were added to *Supplementary Table 4*, where none of the outcomes tested were significantly influenced by the proportion of cardiac arrest patients.
- The Results section was amended to state: "*Lastly, the proportion of cardiac arrest patients did not significantly impact all-cause mortality, whether at 30 days or at the longest available follow-up*."
- Importantly, none of the safety outcomes could be assessed in this meta-regression due to the limited number of available studies.
- As already mentioned in the Limitations section for other meta-regressions (AMI-CS and MCS), these findings should be interpreted with caution given the small number of included studies, and are presented primarily for exploratory purposes.

Reviewer #2: Dear authors,
Dear editor,
The current manuscript aims to summarize the current evidence on cooling for cardiogenic shock in the form of a meta-analysis. The topic is of interest and the approach was rigourous. The manuscript reads fluid. The conclusions are supported by the findings and nuanced.
I'm generally not a big fan of meta-analysis. But in this case it makes sense because there are a few RCT's that were all very small.
I have minor comments:
-Maybe in the figure legends, a little bit more explanation might be appropriate.

We thank the reviewer for his/her appreciation of our manuscript. In order to enhance readability, we have added more detailed explanations to the figure legends, especially for the trial sequential analyses, which are now presented as follows:

- Figure 1. Study selection.
- Figure 2. Risk of bias assessment using the RoB2 and ROBINS-I tools: “*Upper panel shows risk of bias assessment for randomized controlled trials using the Cochrane Risk of Bias 2 (RoB 2) tool across five domains. Lower panel shows risk of bias assessment for observational studies using the ROBINS-I tool across seven domains.”*
- Figure 3. Meta-analysis of efficacy and safety endpoints for hypothermia in cardiogenic shock patients: “*A represented the main analysis. B represents the sensitivity analysis conducted using the incidence rate of outcomes per person-years to account for varying follow-up durations across the trials*.”
- Figure 4. Sensitivity analysis of efficacy and safety endpoints for hypothermia in cardiogenic shock patients: “*A represents the sensitivity analysis performed after excluding the HYPO-ECMO trial. B represents the sensitivity analysis performed including only low risk bias studies*.”
- Figure 5. Trial sequential analysis of 30-day all-cause mortality outcome: “*The blue Z-curve represents the cumulative evidence from included studies plotted against the cumulative sample size. The horizontal red lines represent conventional boundaries for statistical significance (α = 0.05), while the slanted red lines show the trial sequential monitoring boundaries (DeMets boundaries) and futility boundaries. The required information size was calculated as 4,356 patients assuming a 10% relative risk reduction, 5% type I error, and 90% power*.”

- How was MCS defined for the sensitivity analysis? ECMO might differ from other forms since it is possible to cool very efficiently and rapidly...
We apologize for the unclear definition. Here, MCS was defined as the use of any temporary mechanical circulatory support device among Impella, IABP, or ECMO. We fully agree with the reviewer that ECMO represents a specific entity among MCS modalities, as it enables particularly efficient and rapid cooling, as correctly noted.

As we previously detailed in response to another reviewer, targeted temperature achievement was generally good across all included studies, regardless of whether ECMO circuits or other devices were used for cooling.

That said, as shown in *Table 1*, nearly all patients categorized under MCS in our meta-analysis actually received ECMO (337 out of 346), further supporting the relevance of a specific analysis on this subgroup. Therefore, to address the reviewer’s concern as thoroughly as possible, we conducted an additional meta-regression focused solely on ECMO use.

As a result:

- We refined the terminology by specifying "temporary" before MCS throughout the manuscript and tables
- We have clarified this point in the Methods section: "*Eventually, in light of gaps in the evidence and previous studies (1, 7–13), a meta-regression analysis was conducted to assess the effect of AMI-CS, temporary MCS (Impella or intra-aortic balloon pump [IABP] or extracorporeal membrane oxygenation [ECMO]), and cardiac arrest on mortality*."
- We have specified and added the results to *Supplementary Table 4*: “*Compared to overall temporary MCS, ECMO alone was also associated with improved survival, although it did not reach statistical significance for reduction in bleeding risk (p = 0.09). This could reflect either an intrinsically higher bleeding risk with ECMO or limited power due to the small number of studies available*.”
- We have revised the corresponding Results paragraph as follows: "*Conversely, increased MCS utilization was associated with enhanced hypothermia benefits, showing improved 30-day survival (OR 0.48 [0.44–0.51], p<0.01) and reduced bleeding risk (OR 0.54 [0.37–0.77], p<0.01). A similar trend was observed in the ECMO-only subgroup, although the reduction in bleeding risk did not reach statistical significance (p = 0.09)."*
- As previously stated, these findings should be interpreted with caution given the small number of studies included, and are presented primarily for exploratory purposes, as noted in the Limitations section.

Reviewer #3: 4 RCT and 3 non-randomized (case-control) studies on hypothermia in patients with cardiogenic shock have been included in this metaanalysis, involving 338 patients in the hypothermia group and 357 in the control group. The authors observed a lack of difference regarding the primary outcome (30-day mortality) but a significant mortality difference at the longest follow-up available, a finding that is not confirmed by 3 different sensitivity analyses.  There was no significant difference between the two groups regarding the safety outcomes. The authors conclude that mild hypothermia is beneficial in patients with cardiogenic shock, although the evidence is not robust.
According to PROSPERO, the primary outcome of this metaanalysis was supposed to be "30-day" mortality.  In the manuscript, it has been transformed into "all-cause" mortality, without precision regarding the timing…  I believe that it should remain 30-day mortality and that the overall result should be presented as negative. The positive difference at 1-year must be considered as an incidental random finding.  This is confirmed by the 3 sensitivity analyses (all negative), especially the one excluding the studies with a high risk of bias. The trial sequential analysis result fall short of significance, and therefore this finding must be presented as a lack of difference rather than "a trend toward improvement".
We thank the reviewer for his/her insightful comments regarding our manuscript, which prompted us to thoroughly revise it.
As highlighted and requested by the Editor, we have entirely restructured the manuscript in line with the framework suggested by the reviewer.

As correctly noted, our PROSPERO registration listed 30-day all-cause mortality as the primary outcome. However, following extensive discussions within our scientific committee, we initially chose to prioritize all-cause mortality at the longest follow-up duration in the manuscript. This decision was based on three main considerations:

- Indeed, as mentioned, studies such as Danger-Shock have demonstrated that early interventions in CS, even including short-duration treatments, may lead to delayed benefits through improved myocardial recovery, thereby influencing long-term outcomes
- From a methodological standpoint, more data were available at the longest follow-up, which was deemed particularly relevant given the limited number of studies in the field of hypothermia in CS
- Other large-scale, authoritative studies referenced in hypothermia guidelines, such as the TTM2 trial, have also evaluated medium- to long-term mortality, typically at 6 months.

That said, we fully acknowledge the clinical relevance of 30-day mortality, especially in the acute setting of CS, and agree that it should be prioritized in line with our original study registration.

Accordingly, we have substantially revised the manuscript to designate 30-day all-cause mortality as the primary outcome, and all-cause mortality at the longest follow-up as a secondary outcome. Below are the key changes implemented in the revised manuscript:

- Abstract – Results: “*Hypothermia was associated with improved survival at the longest available follow-up (OR 0.79 [0.62–0.99]), but not at 30 days (OR 0.83 [0.54–1.26]). Sensitivity analysis adjusted for follow-up duration confirmed a trend toward reduced all-cause mortality (IRR 0.85 [0.72–1.01]).*” → replaced with: “*Hypothermia was not associated with a significant reduction in all-cause mortality at 30 days (OR 0.83 [0.54–1.26]) or at the longest available follow-up (IRR 0.85 [0.72–1.01]).*”
- Abstract – Conclusion: “*In this meta-analysis, therapeutic hypothermia appeared to be safe and was associated with a potential reduction in all-cause mortality in patients with CS, albeit with very low level of evidence*.” → replaced with: “*In this meta-analysis, therapeutic hypothermia appeared safe but failed to show a significant reduction in all-cause mortality in patients with CS, albeit with very low certainty of evidence. Larger RCTs are warranted to clarify its clinical utility*.”
- Outcomes Section: “*The primary outcome was all-cause mortality. Secondary outcomes included rates of pneumonia, sepsis, bleeding, and supraventricular or ventricular arrhythmias. Each study's definitions of these events were used in the analysis, and events reported at the longest follow-up time point were extracted*.” → replaced with: “*The primary outcome was 30-day all-cause mortality. Secondary outcomes included all-cause mortality at the longest reported follow-up, as well as the incidence of pneumonia, sepsis, bleeding, and supraventricular or ventricular arrhythmias. For each outcome, the definitions provided in the original studies were used in the analysis*.”
- TSA Methods: we clarified: “*The TSA was conducted for the primary outcome of 30-day all-cause mortality and for all-cause mortality at the longest reported follow-up to assess the robustness of our findings*.”
- Results Section: The paragraph starting with “*Figure 3A shows that therapeutic hypothermia was associated with a significant reduction*…” → replaced with: “*Figure 3A shows that therapeutic hypothermia was not associated with a significant reduction in all-cause mortality at 30 days (OR 0.83 [0.54–1.26], I² = 0%, τ² = 0, PI 0.39–1.75). At the longest follow-up, a modest reduction was observed (OR 0.79 [0.62–0.99]), with low heterogeneity, though the PI included both benefit and neutrality (0.52–1.19). Conversely, sensitivity analysis adjusted for follow-up duration revealed the absence of a statistically significant reduction (IRR 0.85 [0.72–1.01]*”
- TSA Results Section: Previously: “*TSA for the primary outcome of all-cause mortality showed a trend toward improvement…*” → replaced with: “*TSA for the primary outcome of 30-day all-cause mortality showed no statistically significant difference between therapeutic hypothermia and control groups, as the Z-curve did not cross the conventional boundary (Figure 5). The Z-curve also did not cross the futility boundary or reach the required information size (n = 4356), indicating that the current evidence is underpowered to detect or exclude a clinically relevant difference. Similarly, TSA for all-cause mortality at the longest follow-up confirmed the absence of a statistically significant benefit, with the Z-curve crossing neither the conventional nor futility boundaries and not reaching the required information size (Supplementary Figure 2). Both analyses indicate that current evidence is insufficient to demonstrate a benefit of therapeutic hypothermia in CS patients, and substantially more data would be needed to draw definitive conclusions*.”
- Main Discussion Points: point 1 and point 4 revised as follows: *1) Based on currently available data, therapeutic hypothermia is not associated with a statistically significant reduction in all-cause mortality in CS, although the certainty of evidence is very low*; *4) Despite a strong physiological rationale and encouraging preclinical data, prospective clinical studies remain scarce, and further research is still warranted to clarify the potential role of hypothermia in this setting*.
- Discussion – Interpretation: Previously: “*our main analysis found that therapeutic hypothermia was associated with a significant 21% reduction…*” → replaced with: “*In our main analysis, therapeutic hypothermia failed to show a significant reduction in 30-day all-cause mortality. Similarly, no improvement in overall survival was observed at the longest follow-up in sensitivity analyses or after adjustment using incidence rate ratios*.”
- Discussion – Limitations: “*The lack of robustness in the observed benefit*…” → replaced with: “*The lack of observed survival benefit may also result from insufficient statistical power, due to the limited number of studies and small sample sizes*.”
- Discussion – General Interpretation: “*Our study illustrates that although hypothermia showed encouraging results in pre-clinical CS studies*…” → replaced with: “*Overall, our study illustrates that, although hypothermia has shown encouraging results in preclinical CS studies, there is currently insufficient data to support its effectiveness in clinical practice. However, at this stage of research, it is still too early to formally conclude its inefficacy, given the limited number of studies conducted, as highlighted by the results of the trial sequential analysis*”
- Conclusion (Full Text): “*In this meta-analysis, therapeutic hypothermia appeared safe but failed to show a significant reduction in all-cause mortality in patients with CS, albeit with a very low certainty of evidence. Given the limited number of studies, small sample sizes, and inconclusive TSA results, it remains premature to draw definitive conclusions regarding either efficacy or inefficacy. Larger, well-designed RCTs are still needed to clarify the role of therapeutic hypothermia in this critically ill population*.”
- Figures and Supplementary Materials: We reorganized *Figures 3* and *4* and *Supplementary Table 5* to reflect 30-day all-cause mortality as the primary outcome. We added *Figure 5* for the TSA on 30-day mortality and moved the TSA on longest follow-up to *Supplementary Figure 2*. We chose to retain both TSA analyses, as the longest follow-up includes more patients and could have yielded different insights. However, the similar findings between the two strengthen the consistency and robustness of our results.

In conclusion, we hope these substantial revisions clarify our interpretation and reinforce our commitment to methodological rigor. Despite encouraging preclinical data, therapeutic hypothermia failed to demonstrate a clinical benefit in CS based on currently available data. Nevertheless, our study highlights a critical knowledge gap and provides a strong rationale for the conduct of larger-scale clinical trials in this area.

The limitation chapter is too long and not focused on limitations.  Part of it belongs to the discussion.

We apologize for the confusion. As suggested by the reviewer, we have shortened the “Limitations” section by moving several interpretative elements to the “Discussion” section. Specifically, the following points were transferred to the Discussion: (1) the hypothesis that the prevention of hyperthermia, rather than strict hypothermia, might be the key therapeutic target; (2) the need to tailor the duration and application of hypothermia according to the heterogeneous etiologies and phenotypes of cardiogenic shock; and (3) the discussion on the impact of sedation, mechanical ventilation, and shivering on the feasibility and physiological effects of hypothermia. The revised Limitations section now focuses on methodological and structural limitations only, such as the heterogeneity in hypothermia protocols, inconsistent reporting of temperature management details, the limited number of events in safety analyses, and the absence of data on withdrawal of life-sustaining therapy protocols.

The conclusion should be "negative" to be supported by the results.

In line with the reviewer’s recommendation and to ensure that our conclusions remain strictly supported by the data, we have revised the general conclusion to adopt a more neutral and “negative” tone. The updated conclusion now states: "*In this meta-analysis, therapeutic hypothermia appeared safe but failed to show a significant reduction in all-cause mortality in patients with CS, albeit with a very low certainty of evidence. Given the limited number of studies, small sample sizes, and inconclusive results from trial sequential analyses, it remains premature to draw definitive conclusions regarding either efficacy or inefficacy. Larger, well-designed RCTs are still needed to clarify the role of therapeutic hypothermia in this critically ill population*."
